# Rethinking the Origin of Primates by Reconstructing Their Diel Activity Patterns Using Genetics and Morphology

**DOI:** 10.1038/s41598-017-12090-3

**Published:** 2017-09-19

**Authors:** Yonghua Wu, Haifeng Wang, Haitao Wang, Elizabeth A. Hadly

**Affiliations:** 1 School of Life Sciences, Northeast Normal University, 5268 Renmin Street, Changchun, 130024 China; 2 Jilin Provincial Key Laboratory of Animal Resource Conservation and Utilization, Northeast Normal University, 2555 Jingyue Street, Changchun, 130117 China; 3 Department of Bioengineering, Stanford University, Stanford, California, 94305 USA; 4 Jilin Provincial Engineering Laboratory of Avian Ecology and Conservation Genetics, Northeast Normal University, 5268 Renmin Street, Changchun, 130024 China; 5 Department of Biology, Stanford University, 371 Serra Mall, Stanford, CA 94305-5020 USA

## Abstract

Phylogenetic inference typically invokes nocturnality as ancestral in primates; however, some recent studies posit that diurnality is. Here, through adaptive evolutionary analyses of phototransduction genes by using a variety of approaches (restricted branch/branch-site models and unrestricted branch-site-based models (BS-REL, BUSTED and RELAX)), our results consistently showed that ancestral primates were subjected to enhanced positive selection for bright-light vision and relatively weak selection for dim-light vision. These results suggest that ancestral primates were mainly diurnal with some crepuscularity and support diurnality as plesiomorphic from Euarchontoglires. Our analyses show relaxed selection on motion detection in ancestral primates, suggesting that ancestral primates decreased their emphasis on mobile prey (e.g., insects). However, within primates, the results show that ancestral Haplorrhini were likely nocturnal, suggesting that evolution of the retinal fovea occurred within ancestral primates rather than within haplorrhines as was previously hypothesized. Our findings offer a reassessment of the visual adaptation of ancestral primates. The evolution of the retinal fovea, trichromatic vision and orbital convergence in ancestral primates may have helped them to efficiently discriminate, target, and obtain edible fruits and/or leaves from a green foliage background instead of relying on mobile insect prey.

## Introduction

Primates are vision-orientated and are distinguished from other mammals by their relatively enhanced visual system, including unique trichromatic colour vision^1^ and relatively high degrees of orbital convergence^2–5^. This visual specialization is likely to be strongly influenced by the specific diel activity pattern of ancestral primates (e.g., nocturnality or diurnality), the origin and timing of which continue to be debated^6^. Extant primates span various diel activity patterns ranging from nocturnal prosimians to diurnal anthropoids, with a few species (e.g., lemurs) being cathemeral, with activity unconfined to a particular time of day or night^7^. This variation across the primates suggests several independent evolutionary origins of diel activity patterns, but there is not consensus about which activity pattern is ancestral, and which is derived^8^. Ancestral primates have long been considered nocturnal based on parsimonious inference from their phylogeny^3,6,9,10^, which is also supported by Bayesian or maximum likelihood reconstruction of their diel activity patterns^8,11^. In the context of the visual predation hypothesis, one of several hypotheses for the origin of primates, the presumed nocturnality of ancestral primates is thought to have preceded the morphological convergence of their orbits. This hypothesis suggests that orbital convergence may have resulted from the nocturnal predatory adaptations of ancestral primates while in pursuit of insects^12,13^. However, recent studies challenge the plesiomorphy of nocturnality, favouring the possibility of diurnality in ancestral primates despite controversy^14^. In particular, the inference of trichromatic colour vision in ancestral primates suggests that early primates were diurnal or cathemeral^15,16^. Lending further support to this hypothesis is fossil evidence. *Teilhardina asiatica*, which is phylogenetically near the root of Primates, shows significantly convergent but small orbits, supporting diurnality in ancestral primates^17^. Evidence endorses both the nocturnality and the diurnality hypotheses, and thus the diel activity pattern of ancestral primates remains unclear.

In vision, light is converted into electrical signals by the phototransduction pathway in retinal photoreceptor cells, including cones and rods. Cones and rods are functionally active in different light conditions. Cones are specialized for detecting bright light, and rods are specialized for detecting dim light. The functional specializations of cones and rods are due to the activation of different vision-related genes^18^. Though cones and rods use different genes, the phototransduction cascade in both begins with the activation of opsins by light. The activated opsins subsequently activate their downstream proteins involved in the phototransduction pathway and eventually hyperpolarize cells (Supplementary Fig. 1). Along with the activation of these proteins, their inactivation is believed to be important for subsequent photoresponse recovery^19^. A timely photoresponse recovery helps photoreceptors respond to subsequently absorbed photons and is essential for high temporal resolution, which is thought to be critical for motion detection^20^.

Two recent studies show that differential selection on the cone-expressed genes and rod-expressed genes can reflect different diel activity patterns, as demonstrated by the discrimination of diurnal and nocturnal taxa in reptiles (including birds) and mammals^21,22^. Thus, adaptation to daytime or nighttime activity likely exerts strong selection pressure on the cone-expressed genes and the rod-expressed genes, respectively. Wu *et al*. (2016) demonstrated that the genes contributing to the photoresponse recovery have undergone strong positive selection in falcons, which are aerial hunters with powerful eyesight for detecting motion^23^. Their findings indicated that some vision genes involved in the phototransduction pathway can be used for both reconstructing diel activity and analysing motion-detection ability in vertebrates. Thus, these genes can be used to explore early primate vision and examine the visual predation hypothesis, specifically, the role of the nocturnal predation abilities of ancestral primates in the pursuit of mobile insects^6,12,13^.

In this study, we analysed the adaptive evolution of the phototransduction genes to infer the evolution of the diel activity patterns and motion detection ability of primates using restricted branch/branch-site models implemented in PAML^24^ and unrestricted branch-site-based models (BS-REL, BUSTED and RELAX) available at Datamonkey webserver (http://www.datamonkey.org/)^25^. Our study showed no evidence of a specifically enhanced ability for motion detection in ancestral primates compared with their closest relatives (e.g., tree shrews and colugos); rather, the results suggest relaxed selection for motion detection in ancestral primates. Our results suggest that ancestral primates were likely primarily diurnal, a trait likely retained from ancestral Euarchontoglires. Therefore, our results reject the visual predation hypothesis as an explanation of the origin of visual adaptation in primates. Our reconstruction of the diel activity patterns reveals a more complicated evolutionary pathway of the diel activity patterns within primates than previously thought, and it provides insights into the origins of the highly specialized visual adaptations in primates.

## Results and Discussion

### The diurnality of ancestral primates

**Figure 1:**
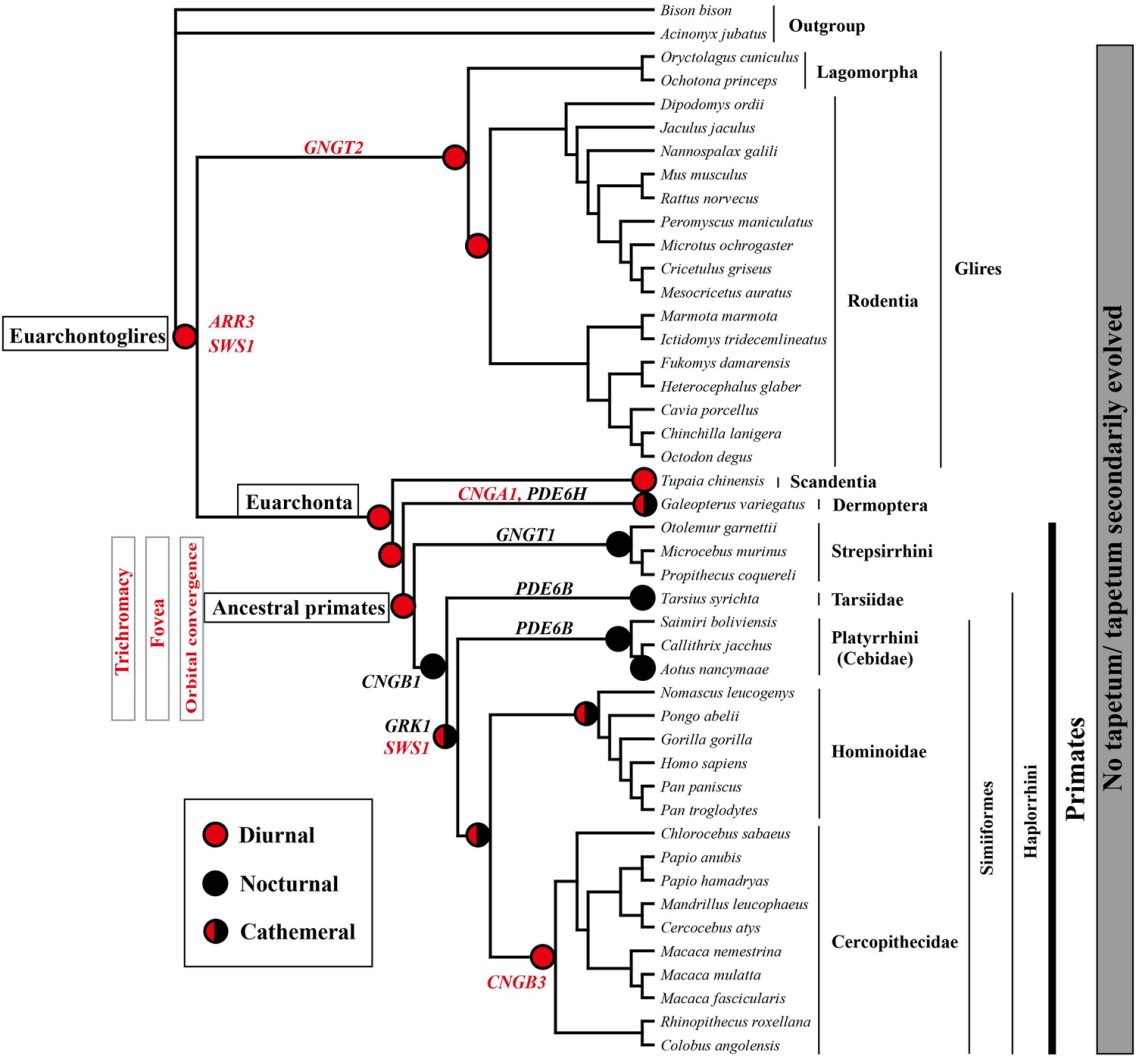
Reconstruction of the diel activity patterns. The diel activity patterns are reconstructed based on the positive selection analyses of the phototransduction genes using the branch-site model of PAML. The positive selection signals of cone-expressed genes (red), rod-expressed genes (black) and both along certain branches are respectively used as an indicator of diurnality, nocturnality and cathemerality. Lack of positive selection signals along certain branches is treated as the retention of the diel activity patterns of their most recent common ancestors. The common ancestor of modern primates is inferred to have retinal fovea, trichromacy, and convergent orbits based on this study and previous studies^6,7,15–17^. The phylogenetic relationships among species follow published studies^63–68^.

**Figure 2:**
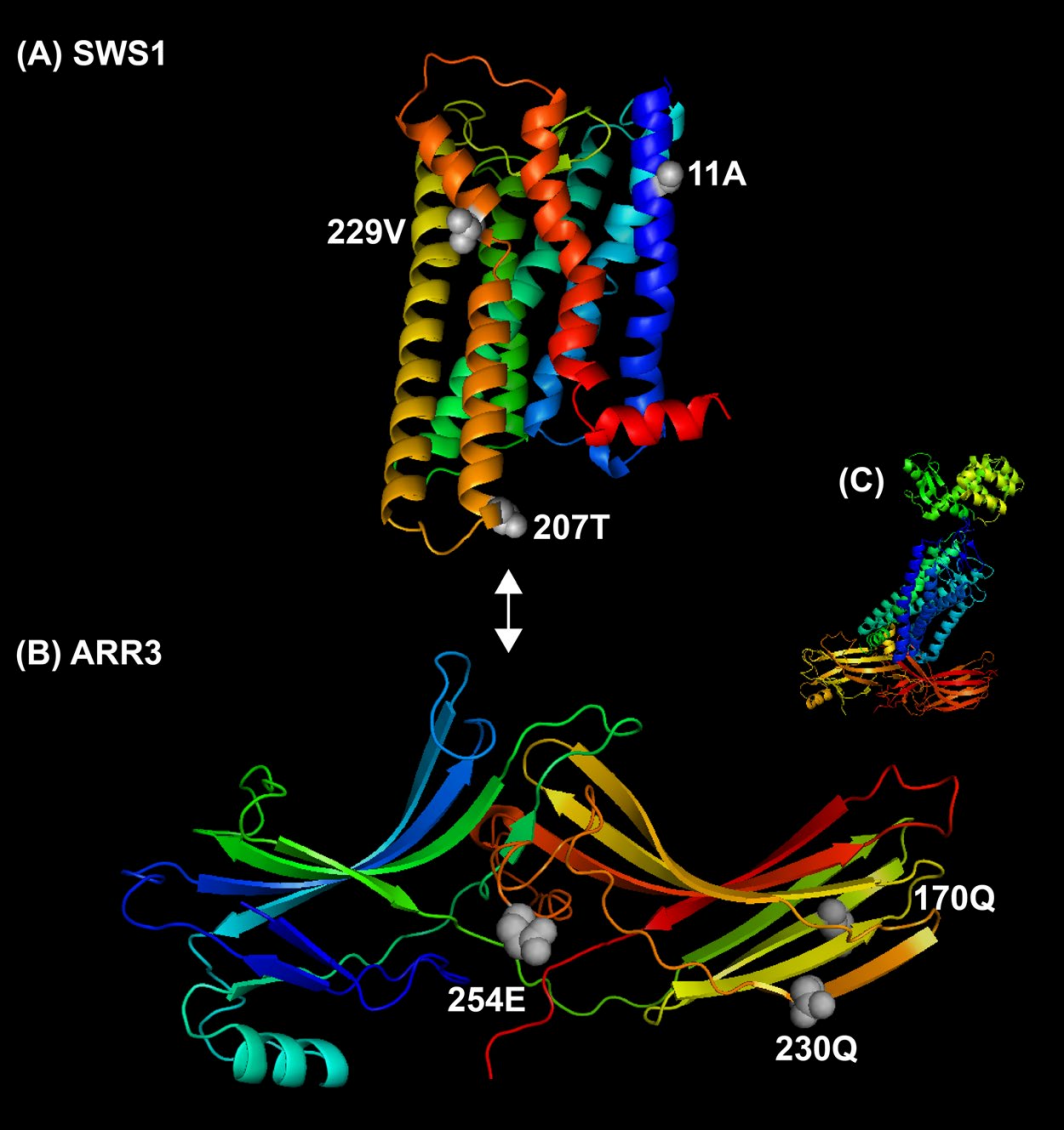
Ancestral protein 3D structures and positively selected sites (grey spheres). Ancestral 3D protein structures of *SWS1* (**A**) and *ARR3* (**B**) were reconstructed using the homology modeling in SWISS-MODEL^71^ based on ancestral protein sequences. The ancestral protein sequences were reconstructed for the most recent common ancestor of extant Euarchontoglires based on the empirical Bayes approach implemented in PAML with JTT substitution model used^69^. The 3D protein structures are visualized and modified using PyMOL (The PyMOL Molecular Graphics System, Version 1.3, Schrödinger LLC, https://www.pymol.org/). Bidirectional arrow shows the putative direction of molecular interaction between *SWS1* and *ARR3* based on the published study that demonstrates the crystal structure of rhodopsin- arrestin complex (**C**)^72^, which are two paralogous proteins of that of *SWS1* and *ARR3*, respectively.

Primates are characterized by high degrees of orbital convergence, which permit stereoscopic vision^2–5^. This characteristic suggests that the vision of ancestral primates might have been subjected to distinct selection, providing an *a priori* hypothesis for our adaptive evolutionary analyses. To determine the possible selection acting on the vision of ancestral primates, we examined the adaptive evolution of 33 vision genes that are known to be involved in the phototransduction pathway in rods and cones (Supplementary Fig. 1, Supplementary Table 1) along branches of interest (Fig. 1, Table 1). Positively selected genes (PSGs) were first analysed using the branch model and the branch-site model as implemented in the Codeml program in PAML^24^. PSGs were only found using the branch-site model, and these PSGs had statistically significant ω values greater than one according to likelihood ratio tests (LRT). Their positive selection signals remained robust when phylogenetic uncertainty and initial value variations of kappa and omega were taken into account. Using PAML, an initial examination of positive selection signals along the ancestral primate branch (foreground branch) showed no PSGs. Subsequently, we searched for signals of positive selection along the branch of Primatomorpha, which includes two sister groups, Primates and Dermoptera, and along the branch of Euarchonta (including Primatomorpha and Scandentia), and we again found no PSGs. Two PSGs, *ARR3* and *SWS1*, were detected along the ancestral branch of Euarchontoglires, which includes two sister groups, Euarchonta and Glires. Both PSGs are genes that are known to be specifically expressed in cones^18,26,27^. *SWS1* encodes the violet/ultraviolet-sensitive cone opsin. *ARR3* encodes the retinal cone arrestin-3, which plays a role in inhibiting activated opsins in cones and contributes to the photoresponse recovery. Behavioural studies in zebrafish have shown that *ARR3* deficiency causes a prolonged photoresponse recovery and reduced temporal resolution^28^. Further analyses identified three positively selected sites for each of the two PSGs (Supplementary Table 2), and all of these positively selected sites showed fixed (or nearly fixed) amino acid differences between Euarchontoglires and its sister taxon, Laurasiatheria (outgroup) (Supplementary Figs 2 and 3). Based on ancestral sequence reconstruction, these positively selected amino acids in Euarchontoglires appear to have been secondarily modified (Supplementary Figs 2 and 3). These positively selected sites are shown mapped on their corresponding protein structures in Fig. 2. In *SWS1*, two positively selected sites, 11A and 229V, are located in the transmembrane region, and 207T is located on the cytoplasmic side (Fig. 2, Supplementary Fig. 4)^29,30^. We found two positively selected sites, 207T of *SWS1* and 254E of *ARR3*, that were close to the contact interface between *SWS1* and *ARR3*. Given the potential interaction between *SWS1* and *ARR3*, we suspect that these two sites might be directly or indirectly involved in the molecular interaction (Fig. 2). The findings of i) the two positively selected cone-expressed genes and ii) the positively selected sites that are possibly involved in their molecular interaction associated with the photoresponse recovery suggest that ancestral Euarchontoglires might have undergone adaptive evolution towards bright-light environments (e.g., daytime) and may have evolved an enhanced capability for motion detection.

**Table 1:** Positively selected genes (PSGs) identified based on the branch-site model of PAML. The PSGs for each of our focal branches are shown with the *P-*values based on the likelihood ratio tests. All PSGs found in this study are based on the branch-site model and no PSGs are detected based on the branch model. For convenience, only the ω values for the foreground branches are shown.

Taxa /Genes	Parameter estimates	2∆L	df	*P*-value
**Euarchontoglires**				
*ARR3*	*p*_0_ = 0.771 *p*_1_ = 0.219 *p*_*2a*_ = 0.008 *p*_*2b*_ = 0.002	4.02	1	0.045
	*ω*_0_ = 0.139 *ω*_1_ = 1.000 ***ω***_***2a***_ ** = 39.887** ***ω***_***2b***_ ** = 39.887**			
*SWS1*	*p*_0_ = 0.803 *p*_1_ = 0.185 *p*_*2a*_ = 0.010 *p*_*2b*_ = 0.002	11.65	1	6.415E-04
	*ω*_0_ = 0.079 *ω*_1_ = 1.000 ***ω***_***2a***_ ** = 90.767** ***ω***_***2b***_ ** = 90.767**			
**Dermoptera**				
*CNGA1*	*p*_0_ = 0.854 *p*_1_ = 0.119 *p*_*2a*_ = 0.024 *p*_*2b*_ = 0.003	7.27	1	0.007
	*ω*_0_ = 0.035 *ω*_1_ = 1.000 ***ω***_***2a***_ ** = 8.016** ***ω***_***2b***_ ** = 8.016**			
*PDE6H*	*p*_0_ = 0.889 *p*_1_ = 0.097 *p*_*2a*_ = 0.012 *p*_*2b*_ = 0.001	4.18	1	0.041
	*ω*_0_ = 0.034 *ω*_1_ = 1.000 ***ω***_***2a***_ ** = 57.671** ***ω***_***2b***_ ** = 57.671**			
**Strepsirrhini**				
*GNGT1*	*p*_0_ = 0.963 *p*_1_ = 0.021 *p*_*2a*_ = 0.016 *p*_*2b*_ = 0.000	6.11	1	0.013
	*ω*_0_ = 0.037 *ω*_1_ = 1.000 ***ω***_***2a***_ ** = 25.434** ***ω***_***2b***_ ** = 25.434**			
**Haplorrhini**				
*CNGB1*	*p*_0_ = 0.914 *p*_1_ = 0.075 *p*_*2a*_ = 0.010 *p*_*2b*_ = 0.001	9.49	1	0.002
	*ω*_0_ = 0.046 *ω*_1_ = 1.000 ***ω***_***2a***_ ** = 164.038** ***ω***_***2b***_ ** = 164.038**			
**Tarsiidae**				
*PDE6B*	*p*_0_ = 0.926 *p*_1_ = 0.072 *p*_*2a*_ = 0.003 *p*_*2b*_ = 0.000	8.44	1	0.004
	*ω*_0_ = 0.033 *ω*_1_ = 1.000 ***ω***_***2a***_ ** = 998.999** ***ω***_***2b***_ ** = 998.999**			
**Simiiformes**				
*GRK1*	*p*_0_ = 0.884 *p*_1_ = 0.106 *p*_*2a*_ = 0.009 *p*_*2b*_ = 0.001	3.89	1	0.048
	*ω*_0_ = 0.046 *ω*_1_ = 1.000 ***ω***_***2a***_ ** = 83.645** ***ω***_***2b***_ ** = 83.645**			
*SWS1*	*p*_0_ = 0.810 *p*_1_ = 0.182 *p*_*2a*_ = 0.007 *p*_*2b*_ = 0.002	6.89	1	0.009
	*ω*_0_ = 0.080 *ω*_1_ = 1.000 ***ω***_***2a***_ ** = 50.380** ***ω***_***2b***_ ** = 50.380**			
**Platyrrhini**				
*PDE6B*	*p*_0_ = 0.927 *p*_1_ = 0.071 *p*_*2a*_ = 0.002 *p*_*2b*_ = 0.000	5.06	1	0.024
	*ω*_0_ = 0.033 *ω*_1_ = 1.000 ***ω***_***2a***_ ** = 119.251** ***ω***_***2b***_ ** = 119.251**			
**Cercopithecidae**				
*CNGB3*	*p*_0_ = 0.691 *p*_1_ = 0.305 *p*_*2a*_ = 0.003 *p*_*2b*_ = 0.001	7.78	1	0.005
	*ω*_0_ = 0.124 *ω*_1_ = 1.000 ***ω***_***2a***_ ** = 212.089** ***ω***_***2b***_ ** = 212.089**			
**Aotus**				
*RCVRN*	*p*_0_ = 0.899 *p*_1_ = 0.096 *p*_*2a*_ = 0.005 *p*_*2b*_ = 0.001	3.88	1	0.049
	*ω*_0_ = 0.047 *ω*_1_ = 1.000 ***ω***_***2a***_ ** = 528.256** ***ω***_***2b***_ ** = 528.256**			
**Glires**				
*GNGT2*	*p*_0_ = 0.874 *p*_1_ = 0.083 *p*_*2a*_ = 0.039 *p*_*2b*_ = 0.004	4.52	1	0.033
	*ω*_0_ = 0.098 *ω*_1_ = 1.000 ***ω***_***2a***_ ** = 649.632** ***ω***_***2b***_ ** = 649.632**			

2∆L: twice difference of likelihood values between two nested models; df: degrees of freedom; *p*: proportion of sites in different site classes. The four site classes (*p*_0_, *p*_1_, *p*_*2a*_ and *p*_*2b*_) of the branch-site model are shown.

The adaptive evolution of ancestral Euarchontoglires towards bright-light environments suggests the emergence of daytime activity. If so, we would infer that the other three groups, including ancestral Euarchonta, ancestral Primatomorpha and ancestral primates, were all diurnal as no PSGs were found along their corresponding branches (Fig. 1), as mentioned above. The absence of PSGs suggests that changes in vision-gene function were unlikely and that the three groups might have retained the diurnal activity of ancestral Euarchontoglires. Intriguingly, our examination of positive selection along the branch leading to an extant diurnal tree shrew (*Tupaia chinensis*) showed no PSGs based on PAML results (Fig. 1), suggesting that the modern *T. chinensis* might retain the diel activity pattern of ancestral Euarchontoglires as well. If this is true, it suggests that all four groups under investigation, including ancestral Euarchontoglires, ancestral Euarchonta, ancestral Primatomorpha and ancestral primates, are likely to have had a *T. chinensis*-like diel activity pattern and a *T. chinensis*-like vision ability for motion detection. Unlike *T. chinensis*, two PSGs, including one rod-expressed gene, *CNGA1*, and one cone-expressed gene, *PDE6H*, were detected along the branch leading to the modern Sunda colugo (*Galeopterus variegatus*) (Fig. 1, Table 1). This finding suggests further evolutionary modification towards adaptation to an activity pattern in both nighttime and daytime. *Galeopterus variegatus* is typically nocturnal, but daytime activities have been reported^31,32^, supporting our molecular results. We also examined the adaptive evolution of the vision genes along the ancestral Glires branch and the ancestral Rodentia branch. One cone-expressed gene, *GNGT2*, was detected as being under positive selection along the ancestral branch of Glires, suggesting the diurnality of this group (Fig. 1, Table 1).

Given the inferred predominant diurnality of ancestral Euarchontoglires, we might expect to find other evidence suggesting diurnality. One such piece of evidence is the relative lack of the tapetum in crown groups of Euarchontoglires compared with Laurasiatheria. The tapetum is a biologic reflective layer in the retina that increases the sensitivity of the retina under dim-light conditions, and it is often absent in diurnal taxa^3,33^. In placental mammals, the lack of tapetum appears to be common in Euarchontoglires compared with its sister group, Laurasiatheria^3,10,34–39^. Among many taxa studied in Euarchontoglires, only two species (*Cuniculus paca* and *Pedetes capensis*) from Rodentia and one of two suborders of Primates, Strepsirrhini, are known to have a tapetum, with all other taxa, including Scandentia, Dermopera and Haplorrhini, not possessing this structure^10,34,36,37,39,40^. Among those groups with a tapetum, the tapetum appears to be unique to each group and distinct from the tapeta of other mammalian taxa (e.g., Laurasiatheria), suggesting the possibility of the independent and secondary evolution of tapeta in these taxa. For example, Strepsirrhini has a tapetum cellulosum, which has a different function than that of the tapetum fibrosum or retinal tapetum in many other groups of Theria^38^. Additionally, the tapetum cellulosum of Strepsirrhini has riboflavin as its active principle, which is apparently unique to this group and suggests that it is secondarily evolved^3,10^. In rodents, *Cuniculus paca* has a tapetum cellulosum, which is a reflective material of rodlets enriched in sulphur and appears to be unique to this species^34,38^. Among 18 rodent species representing all three major rodent clades, *Pedetes capensis* is the only species found to have a tapetum fibrosum^39^, which is also considered to be secondarily evolved^39^. Therefore, the widespread lack of a tapetum in crown groups of Euarchontoglires and the possible secondary evolution of the tapetum in some Euarchontoglires taxa suggest that ancestral Euarchontoglires might have also lacked a tapetum, consistent with the diurnal activity of this group inferred in this study.

Our results from the branch-site model in PAML showed that ancestral primates might have retained the diurnality of ancestral Euarchontoglires. To increase the robustness of our inferences, we employed two additional methods, the branch site-random effects likelihood (BS-REL) method^41^ and the branch-site unrestricted statistical test for episodic diversification (BUSTED)^42^, to detect episodic positive selection along our focal branches using the same dataset as used in PAML (Supplementary Tables 3 and 4). BS-REL and BUSTED are mainly distinguished from the branch-site model of PAML by their assumption that positive selection is allowed on any branch as opposed to being restricted to foreground branches. For our four focal branches, including ancestral Euarchontoglires, ancestral Euarchonta, ancestral Primatomorpha and ancestral primates, BS-REL did not detect any PSGs for any branch (Supplementary Table 4), whereas BUSTED detected PSGs for two branches, including ancestral Euarchontoglires and ancestral primates (Supplementary Table 3). Specifically, BUSTED detected one positively selected cone-expressed gene, *CNGB3*, along the ancestral Euarchontoglires branch, which was not detected by PAML, and BUSTED also detected one cone-expressed gene, *SWS1*, at close to marginal significance (*P* = 0.0505), which was found to be under positive selection by PAML. For the ancestral primate branch, BUSTED detected three PSGs, including two cone-expressed genes, *SWS1* and *PDE6C*, and one rod-expressed gene, *PDE6A*. *PDE6C* and *PDE6A* encode the hydrolytic subunits of cGMP phosphodiesterase (PDE6), which is known to play a critical role in amplifying signals in the phototransduction cascade, in cones and rods, respectively. The two cone-expressed genes, *SWS1* and *PDE6C*, showed stronger positive selection signals (both LRT *P* values were close to 0.01) than did the rod-expressed gene, *PDE6A*, which had an LRT *P* value of 0.03. The strong positive selection for bright-light vision relative to that for dim-light vision suggests a potentially intensified selection for increased visual acuity in adapting to bright-light environments (e.g., daytime) in ancestral primates. In addition, it suggests that ancestral primates might have mainly been diurnally active, with partial activity in dim-light conditions (e.g., crepuscularity), which is consistent with diurnality as inferred from PAML. The predominant diurnality of ancestral primates might have been retained from ancestral Euarchontoglires, which was also reconstructed as being diurnal, because only the cone-expressed gene (*CNGB3*) was found to be under positive selection (based on BUSTED; Supplementary Table 3).

Despite our consistent results within Primates with these methods, there were some inconsistencies when comparing results between PAML and BUSTED for the ancestral Glires branch, the ancestral rodent branch and the extant tree shrew (*Tupaia chinensis*) branch (Supplementary Table 3). For example, for the ancestral Glires branch, PAML detected only one positively selected cone-expressed gene (*GNGT2)*, whereas BUSTED detected two additional PSGs: another cone-expressed gene (*GRK7*) and one rod-expressed genes (*SLC24A1*). Similarly, for the ancestral rodent branch and the tree shrew branch, PAML did not identify any PSGs, whereas BUSTED identified three PSGs (*CNGA3, RCVRN* and *PDE6B*) and one PSG (*PDE6A*), respectively. The relative reliabilities of the two methods are unknown since they have different underlying assumptions, and the extent to which these assumptions match our real data remains unknown. However, two recent studies found that the PAML results were consistent between the evolution of the phototransduction genes and diel activity patterns^21,22^. Therefore, here we chose to reconstruct the diel activity patterns based on the PAML results (Fig. 1).

The diurnality of ancestral primates is supported by both the branch-site model of PAML and BUSTED. The diurnality of ancestral primates was originally proposed based on the widespread distribution of polymorphic trichromatic colour vision in both of the extant primate groups (Strepsirrhini and Haplorrhini)^15,36,43^, suggesting trichromacy in stem-primates^16^. In Eutheria, trichromatic colour vision appears to be unique to primates^1^, and it is absent even in the close relatives of Primates (Glires, Scandentia and Dermoptera), in which either dichromatic or monochromatic colour vision has been reported^37,44–46^. Trichromatic colour vision is considered to help primates discriminate red–green colours and would thus be advantageous for the detection of ripe fruits and young leaves against a green foliage background^47–49^. Additional evidence in favour of the diurnality of ancestral primates comes from the reconstruction of the diurnality of early primate fossils^7,17^, including the known earliest euprimate fossil, *Teilhardina asiatica* ^17^. Moreover, the hypothesized evolution of the tapetum within Strepsirrhini, as described above, and the absence of tapetum in its sister clade, Haplorrhini, and in the outgroups of Primates is consistent with ancestral primates lacking a tapetum and being diurnal. Thus, we conclude that the diurnality of ancestral primates is currently supported by the weight of evidence including trichromacy, the inferred diurnality of early euprimate fossils, the lack of tapetum and the molecular results of the present study.

### Intensified selection for bright-light vision and relaxed selection for motion detection in ancestral primates

To investigate selection for diurnality, which we infer from our analysis of cone-expressed genes, we used RELAX^50^ to evaluate the selection intensity parameter (*k*) and its statistical significance across all 33 phototransduction genes within ancestral primates. To test for selection in ancestral primates, we used all other non-primate branches within Euarchontoglires as the reference branches (Table 2). Among the 33 genes studied in stem-primates, no rod-expressed genes were found to be under either relaxed selection or intensified selection, whereas among the cone-expressed genes, intensified selection was detected for two cone-expressed genes, *SWS1* and *CNGB3*, with highly statistically significant *k* values (*k* > 1, P < 0.01) (Table 2). Specifically, for *SWS1*, we found that 95% of the sites that were under relatively moderate purifying selection in the non-primate branches within Euarchontoglires were subjected to stronger purifying selection in the ancestral primate branch (ω = 0.08 shifting to ω = 0.0000445). In addition, 4.7% of sites that were under relatively weak positive selection in the reference branches became subjected to more intensified positive selection within ancestral primates (ω = 2.45 shifting to ω = 35.0). *CNGB3* showed a similar trend (Table 2). Therefore, our results showed that there was no relaxed selection for either the cone-expressed genes or the rod-expressed genes, whereas intensified selection for bright-light vision was found in ancestral primates. In addition, we analysed the photoresponse recovery genes, which determine the temporal resolution of images moving across the field of view and hence provide motion detection^20^. Intriguingly, we detected two photoresponse recovery genes, *RCVRN* and *RGS9*, which are known to be involved in the inactivation of opsins and transducin, respectively, to be subjected to significant relaxed selection (*k* < 1, P < 0.01) in ancestral primates compared with other non-primate Euarchontoglires (Table 2). Both the ω values of the purifying selection and the positive selection of the reference branches were found to become convergent to neutrality (ω = 1) in the ancestral primate branch, suggesting relaxed selection on the photoresponse recovery, indicating that the motion detection ability of primates became less advantageous.

**Table 2:** The genes under the relaxed selection (*k*<1) and the intensified selection (*k*>1) in ancestral primates. The ancestral primate branch was used as test branch and all other non-primate branches within Euarchontoglires were used as reference branches. The value of the selection intensity parameter (*k*) and its statistical significance were calculated using RELAX^50^.

Gene	Model	log L	# par.	Branch set	ω1	ω2	ω3	*K*	*P*-value
***SWS1***									
	Null	−8498.30	144	Reference branch	0.107 (96%)	0.113 (2.3%)	5.30 (2.1%)		
				Test branch	0.107 (96%)	0.113 (2.3%)	5.30 (2.1%)	3.97	0.0011**
	Alternative	−8492.94	145	Reference branch	0.0800 (95%)	0.0830 (0.37%)	2.45 (4.7%)		
				Test branch	0.0000445 (95%)	0.0000514 (0.37%)	35.0 (4.7%)		
***CNGB3***									
	Null	−15533.11	136	Reference branch	0.00 (71%)	0.713 (0.0%)	1.40 (29%)		
				Test branch	0.00 (71%)	0.713 (0.0%)	1.40 (29%)	7.64	0.0013**
	Alternative	−15527.91	137	Reference branch	0.00 (71%)	0.000100 (0.10%)	1.38 (29%)		
				Test branch	E-20 (0.10%)	0.00 (71%)	11.8 (29%)		
***RCVRN***									
	Null	−4812.50	150	Reference branch	0.0745 (88%)	0.0867 (12%)	3.63 (0.66%)		
				Test branch	0.0745 (88%)	0.0867 (12%)	3.63 (0.66%)	0.00	0.0061**
	Alternative	−4808.75	151	Reference branch	0.0713 (89%)	0.0744 (11%)	3.38 (0.74%)		
				Test branch	1.00 (0.74%)	1.00 (11%)	1.00 (89%)		
***RGS9***									
	Null	−9341.51	146	Reference branch	0.0374 (2.3%)	0.0376 (98%)	12.8 (0.068%)		
				Test branch	0.0374 (2.3%)	0.0376 (98%)	12.8 (0.068%)	0.41	0.0029**
	Alternative	−9337.06	147	Reference branch	0.0360 (98%)	0.0366 (2.3%)	14.3 (0.064%)		
				Test branch	0.255 (98%)	0.256 (2.3%)	2.99 (0.064%)		

log L, log-likelihood values, # par., the number of parameters, **P<0.01.

### The evolutionary pathway of the diel activity patterns within primates

We analysed the adaptive evolution of vision genes along the branches of interest within primates and reconstructed the diel activity patterns of primates based on the PAML results. Extant primates are grouped into two groups: Strepsirrhini (lemurs, lorises and galagos) and Haplorrhini (tarsiers, monkeys and apes). Most extant strepsirrhine primates are nocturnal^10^, and we found one rod-expressed gene, *GNGT1*, to be under positive selection along the ancestral strepsirrhine branch (Fig. 1, Table 1). Selection for dim-light vision within ancestral strepsirrhines suggests that this group achieved nocturnality subsequent to its divergence from its diurnal primate ancestors, consistent with other previous studies^8–11^ (Supplementary Fig. 5). Haplorrhini includes the nocturnal Tarsiidae and the mostly diurnal Simiiformes (diurnal except for the nocturnal night (or owl) monkeys in the genus *Aotus* ^10^). Previous studies generally consider ancestral haplorrhines to be diurnal^10,11,16^; however, our molecular results revealed one rod-expressed gene, *CNGB1*, which encodes the β subunit of cyclic nucleotide-gated channel in rod photoreceptors, to be under positive selection along the ancestral haplorrhine branch (Fig. 1, Table 1). Selection for *CNGB1* along this branch, which is also supported by one previous study^51^, suggests that ancestral haplorrhines were nocturnally active, which is consistent with previous results that are based on either parsimonious or maximum likelihood reconstructions^8,9^ (Supplementary Fig. 5). We also explored whether there was positive selection along the branch leading to the extant Philippine tarsier (*Tarsius syrichta*), which is nocturnal. We found positive selection on one rod-expressed gene, *PDE6B*, which encodes the hydrolytic subunit of cGMP phosphodiesterase (Fig. 1, Table 1). Apparently, the nocturnality of the Philippine tarsier is retained from that of ancestral haplorrhines. Given the long-term maintenance of nocturnality in tarsiers since they diverged from the rest of the haplorrhines, their trichromatic vision^36^ is probably retained from that of their diurnal ancestral primates.

Simians are known to include two groups: Platyrrhini (New World monkeys) and Catarrhini (including Old World monkeys, apes and humans). The positive selection analyses along the ancestral simian branch revealed two PSGs, consisting of one rod-expressed gene, *GRK1*, and one cone-expressed gene, *SWS1* (Fig. 1, Table 1), suggesting activity in both dim-light and bright-light conditions. Hence, ancestral simians might have been cathemeral, which is inconsistent with previous inferences of diurnality that are based on parsimonious or maximum likelihood reconstructions^8,9^ (Supplementary Fig. 5). Alternatively, ancestral simians might have undergone independent and separate adaptations to dim-light (e.g., nocturnal) and bright-light (e.g., diurnal) environments at different periods during their evolution rather than at the same time (cathemeral); however, we cannot distinguish these two possibilities here. Either way, our results suggest at least partial nocturnal activity within ancestral simians, which is inconsistent with previous inferences of their diurnality^8,9^. Thus, our results imply that the molecular approach that we used could have the power to reveal a hidden history of trait evolution. Moreover, given our reconstruction of the nocturnality of ancestral haplorrhines, the inferred partial activities of ancestral simians in bright-light conditions implies that ancestral simians might have already started to invade a diurnal niche before becoming fully diurnal. In support of this, we identified one critical amino replacement, A175S (corresponding to A164S in bovine rhodopsin), of the red-sensitive opsin (*LWS*) along the ancestral simian branch. This replacement is known to lead to a long-wavelength shift by increasing λmax (wavelength of maximal absorption) by 6 nm^30^ (Supplementary Figs 6 and 7, Supplementary Table 5). Interestingly, the long-wavelength shift (increase in λmax of 6 nm) of *LWS* in ancestral simians is opposite to the short-wavelength shift of *LWS* (corresponding to S164A in bovine rhodopsin by reducing λmax by 7 nm) in owls and falcons, which plays a role in the adaptation of these birds to crepuscular activity^21^. The long-wavelength shift of *LWS* in ancestral simians might have helped maximize photon absorption by tuning their *LWS* sensitivities to more abundant, long-wavelength light during daytime compared with crepuscular periods^52^. We also analysed positive selection along different branches within Simiiformes. We found one positively selected rod-expressed gene, *PDE6B*, along the branch leading to Cebidae, within Platyrrhini, implying that ancestral Cebidae might have been nocturnal. Intriguingly, except for one photoresponse recovery gene, *RCVRN*, no PSGs were detected further along the branch of the night monkey (*Aotus nancymaae*), suggesting that this species might have retained nocturnality from the ancestral Cebidae (Fig. 1). Because the other two groups of platyrrhines, the families Atelidae and Pitheciidae, were not incorporated in this study, future studies including these two groups would be valuable in reconstructing the diel activity pattern of ancestral platyrrhines.

We also explored selection for differential visual activity within Catarrhini, which includes Cercopithecidae (Old World monkeys) and Hominoidae (apes and humans). The initial investigation for positive selection along the branch of the ancestral catarrhines based on PAML showed no PSGs, suggesting the retention of the diel activity pattern (cathemeral) from that of ancestral simians. Similarly, no PSGs were detected along the ancestral Hominoidae branch, suggesting the cathemerality. Our reconstructions of the cathemerality for both ancestral catarrhines and ancestral Hominoidae are inconsistent with previous reconstructions of diurnality^8,9,35^ (Supplementary Fig. 5). Though extant catarrhine species are generally considered to be diurnal, our molecular results imply that these species are capable of being active at night. In support of this, chimpanzees, which are believed to be diurnal, have also been observed to be active at night^7,53^. We found one positively selected cone-expressed gene, *CNGB3*, which encodes the β subunit of cyclic nucleotide-gated channel in cone photoreceptors, along the branch of ancestral Cercopithecidae (Fig. 1, Table 1), suggesting the diurnality of this group, consistent with previous results^8,9^ (Supplementary Fig. 5). The positive selection on the cone-expressed gene, *CNGB3*, supports more enhanced diurnal vision in Cercopithecidae compared with that in its sister group, Hominoidae.

We also used BS-REL and BUSTED to identify PSGs for our focal branches within primates and we found many consistencies with our PAML results. Among all of our focal primate branches (Fig. 1), BS-REL and BUSTED detected PSGs for only four branches: the ancestral haplorrhine branch, the ancestral simian branch, the ancestral catarrhine branch, and the ancestral Cercopithecidae branch. In ancestral haplorrhines, both BS-REL and BUSTED identified strong positive selection in *CNGB1*, which is consistent with the PAML results, and provided further support of the nocturnality of ancestral haplorrhines. In ancestral simians, one cone-expressed gene (*GNB3*) and one rod-expressed gene (*CNGA1*) were found to be under positive selection, suggesting cathemerality as reconstructed by PAML. In the ancestral catarrhine branch, which was reconstructed to be cathemeral based on the PAML results, one cone-expressed gene, *LWS*, was found to be under positive selection by BUSTED. This suggests that ancestral catarrhines were partially active during the day. Finally, BUSTED detected one positively selected rod-expressed gene, *RH1*, along the ancestral Cercopithecidae branch, which suggests the nocturnality of ancestral Cercopithecidae. However, all extent Cercopithecidae taxa are diurnal, and the nocturnality of ancestral Cercopithecidae as inferred by BUSTED is difficult to explain. Similarly, unexpected results were also noted in the extant diurnal tree shrew branch, in which a rod-expressed gene (*PDE6A*) was identified by BUSTED (Supplementary Table 3). The apparently unexpected results found by BUSTED and BS-REL might partly be due to false positive results with the corrected *P* values > 0.05 (Supplementary Table 3). These results suggest that these programs may have less power to identify positively selected genes compared with PAML, which has revealed positive selection related to phototransduction gene evolution and diel activity pattern variations in birds^21^.

### Insights into the visual adaptations of ancestral primates

Our evolutionary analyses of the genes involved in the timing and sensitivity of vision within Euarchontoglires provide three important insights about the evolution of vision in ancestral primates. **i**) Ancestral primates were predominately diurnal, and their diurnality might have been retained from ancestral Euarchontoglires. **ii**) Ancestral primates might have shifted their reliance from mobile prey (e.g., insects)^5,12^ to immobile food (i.e., plants and/or fruit). **iii**) The retinal fovea is plesiomorphic within primates. Because our results and those of other studies^8,9^ support the nocturnality of ancestral haplorrhines, the timing and selection for the evolution of retinal fovea is in question. The retinal fovea is generally thought to have arisen as an adaptation for high visual acuity in bright-light conditions^54^ and be unique to haplorrhine primates^3^. Because we surmise that ancestral haplorrhines were nocturnal, the emergence of the retinal fovea within this group is difficult to explain. We speculate that the evolution of the retinal fovea might predate ancestral haplorrhines and may possibly extend to ancestral primates, being subsequently retained in haplorrhines and secondarily lost in strepsirrhines. If this is the case, we would expect to detect somewhat rudimentary fovea in strepsirrhines. Intriguingly, the presence of somewhat rudimentary fovea or area centralis is reported in at least three species of the genus *Galago* ^55–58^ and from *Lemur catta* and *Hapalemur griseus* ^59,60^, representing two major clades (Lorisiformes and Lemuriformes) of strepsirrhine primates. The findings of the somewhat rudimentary fovea or area centralis in these strepsirrhine species, which are strikingly similar to that of the nocturnal owl monkey^3,55^, support our hypothesized evolution of the fovea in ancestral primates rather than ancestral haplorrhines.

The possible evolution of the retinal fovea, the morphological evolution of orbital convergence^6,7,17^ and the trichromatic colour vision^15,16^ in ancestral primates provide important insights into the ecological adaptations of ancestral primates, which have resulted in a unique primate-specific visual system, even when compared to their closest outgroup, the tree shrews (to the exclusion of Dermoptera, which are gliding relatives). Compared with the tree shrews, which primarily rely on olfaction for foraging^40^, extant diurnal primates (e.g., simians) have reduced olfaction and are generally regarded as primarily vision-oriented^3^. Such vision-dependency likely also applies to ancestral primates; the earliest diurnal euprimate fossil (*Teilhardina asiatica*) has a significantly shortened snout and very convergent orbits^17^. Traditionally, the evolution of trichromacy in primates has been invoked as being driven by the need to discriminate ripe fruits and/or young leaves from a green background^47–49^. However, we suggest that although trichromacy facilitates the discrimination of edible fruit or leaves, the retinal fovea, which contributes to high visual acuity, could help to target favourite fruits or leaves from a distance. Furthermore, the converged orbits (binocular vision) contribute to stereoscopic vision, which might help to accurately locate target fruits or leaves within a three-dimensional space and provide clearly defined depth perception, allowing the hand to obtain target foods. Therefore, unlike olfaction-dependent tree shrews, vision-oriented ancestral primates might have undergone a complementary and integrated evolution of trichromacy, the fovea, and orbital convergence to help them to efficiently discriminate, target and obtain their favourite food.

## Materials and Methods

### Sequence collection and alignment

We downloaded coding sequences of 33 vision genes that are known to be involved in the rod and cone phototransduction pathway for our focal taxa from GenBank and Ensembl (Supplementary Table 1). Where multiple transcript variants of one gene of a species were available, the longest one was selected; if the longest transcript harboured multiple ambiguous bases (Ns), then the second-longest one was used. We aligned the downloaded sequences of each gene using the online software webPRANK (http://www.ebi.ac.uk/goldman-srv/webprank/)^61^. This software is believed to create a more reliable alignment than that obtained using other software, and it is considered to decrease false positive results in positive selection analyses based on the branch-site model implemented in PAML^62^. The sequence alignments were manually inspected for quality, and sequences that were too short, had low identities or had long indels and/or multiple ambiguous bases (Ns) were removed. After this pruning, only the high-quality alignment results were used for the subsequent analyses.

### Taxa covered

Euarchontoglires includes four groups: Scandentia, Dermoptera, Primates and Glires. In this study, species from all four groups were included. Species of Laurasiatheria, the sister clade of Euarchontoglires, were used as an outgroup. For different genes, the species used were subjected to changes depending on their sequences availabilities (Please see Supplementary Table 1 for details).

#### Primates

In total, 23 primate species from 8 families were used, representing both suborders (Strepsirrhini and Haplorrhini) of Primates. For Strepsirrhini, three families, Galagidae, Indriidae and Cheirogaleidae, were incorporated, which compose the two most divergent clades of Strepsirrhini^63^. For Haplorrhini, five families, Tarsiidae, Cebidae, Hylobatidae, Hominidae and Cercopithecidae, were included, representing all of the four major clades of Haplorrhini^63^.

#### Relatives of primates

*Tupaia chinensis* from Scandentia and *Galeopterus variegatus* from Dermoptera were included.

#### Glires

Glires includes two orders, Rodentia and Lagomorpha. For Lagomorpha, two species, *Ochotona princeps* and *Oryctolagus cuniculus*, representing both of its two families (Ochotonidae and Leporidae), were used. For Rodentia, 18 species from 10 families (Bathyergidae, Caviidae, Chinchillidae, Cricetidae, Dipodidae, Heteromyidae, Muridae, Octodontidae, Sciuridae and Spalacidae) were used, representing all three of its major clades^64^.

#### Outgroup

Species of Laurasiatheria, the sister clade of Euarchontoglires, were used as an outgroup in this study. In most cases, one nocturnal species from Artiodactyla, *Bison bison*, and one diurnal species from Carnivora, *Acinonyx jubatus*, were used. When the sequences of these two species were unavailable or had low qualities, related species such as *Bos Taurus*, *Canis lupus*, *Felis catus* and *Ailuropoda melanoleuca* were alternatively selected.

### Species trees used

Phylogenetic relationships among taxa used in this study followed previous published studies. Specifically, the species relationships among the primates and the phylogenetic relationships among primates, Dermoptera, Scandentia and Glires were in accordance with recently published molecular phylogenies^63,65^. The primate phylogeny^63^ represents a highly resolved phylogeny, and the species relationships are largely consistent with previous results^2^, representing a robust phylogeny. The families relationships within Glires were based on one published mammal phylogeny study^64^, and the species relationships within two families, Cricetidae and Sciuridae, were derived from other sources^66–68^. Regarding the Euarchontoglires phylogeny, it is unclear whether Scandentia is more closely related to Primatomorpha or to Glires^63,64^; therefore, in the present study, we considered both phylogenetic relationships (phylogenetic uncertainty).

### Positive selection analyses using PAML

Following recently published studies that employed the codon-based maximum likelihood methods implemented in the Codeml program in PAML^24^ to determine the consistency between the adaptive evolution of the phototransduction genes and diel activity patterns^21,22^, we used the same method for our positive selection analyses. The maximum likelihood methods estimate the ratio of non-synonymous to synonymous substitutions per site (d_N_/d_S_ or ω) as the indicators of purifying selection (ω < 1), neutral evolution (ω = 1) and positive selection (ω > 1). In this study, based on the species relationships (e.g., Fig. 1) mentioned above, we employed the branch model and the branch-site model for our positive selection analyses. In the analyses, the branches of interest were treated as the foreground branch while all of the other branches were used as background ones. Likelihood ratio tests (LRTs) were then used to compare the null model with the alternative models to determine statistical significance.

#### Branch model

A two-rate branch model was used to identify the branches of interest that were under positive selection. The two-rate branch model allows ω to vary between foreground branches and background branches, and its goodness of fit was compared with the one-rate model, which assumes one single ω value for every branch base on the LRT. When the LRT was statistically significant, the likelihood values of the two-ratio model were then compared with the two-ratio model with a constraint of ω = 1 to examine whether the ω value of the foreground branch was greater than one.

#### Branch-site model

The branch-site model was used to detect positively selected sites along the branches of interest. For the analyses, Test 2, which compares a modified model A with the corresponding null model with the constraint ω = 1, was used. For the modified model A, four classes of sites were respectively assumed. Site class 0 (0 < ω_0_ < 1) and site class 1 (ω_1_ = 1) represent evolutionarily conserved and evolutionarily neutral codons, respectively, in both the background branches and foreground branches, whereas site classes 2a and 2b includes evolutionarily conserved (0 < ω_0_ < 1) or neutral (ω_1_ = 1) codons, respectively, in the background branches, which are allowed to be under positive selection (ω_2_ > 1) along the foreground branches. In the analyses, the Bayes Empirical Bayes method was used to identify positively selected sites.

### Positive selection analyses using BS-REL and BUSTED

We also used two other different methods, the branch site-random effects likelihood (BS-REL) method^41^ and the branch-site unrestricted statistical test for episodic diversification (BUSTED)^42^, to examine the episodic positive selection for our focal branches using the same dataset as that of PAML. BS-REL and BUSTED differ mainly from the branch/branch-site models of PAML in their different assumptions of models. PAML assumes all branches can be partitioned *a priori* into foreground branches and background branches, and only the foreground branches are allowed to undergo positive selection while the background branches are constrained to be negatively selected or neutral (restricted branch/branch-site model). Unlike PAML, BS-REL is unrestricted regarding the occurrence of positive selection across the tree and do not require partitioning branches into foreground and background branches (unrestricted branch-site model). For analyses, BS-REL assumes three ω categories (ω1, ω2 and ω3) representing strong and weak conservation and positive selection for every branch, and then the values of each of the three ω categories with their corresponding site proportions for each branch were calculated. The sequential likelihood ratio testing was used to identify positively selected branches. BUSTED, which is based on the BS-REL model, mainly distinguishes from BS-REL by its capability to test positive selection on particular lineages (interested) without a restriction of occurrence of the positive selection in the rest of the tree. Similarly to PAML, BUSTED normally requires *a prior* partitioning of the tee into the foreground branches (interested) and the background branches and is considered to have an increased power to identify positive selection^42^. The foreground branches are allowed to undergo positive selection (alternative model) or are not allowed to undergo positive selection with ω3 = 1 (null model). And a likelihood ratio statistic is used to determine the fitness of the alternative model. For our analyses, each of all our focal branches was respectively used as the foreground branch and all others were treated as the background branches. Bonferroni multiple testing correction was used to adjust *P* values.

### Robustness tests of positive selection genes

For PAML results, we took effects of the phylogenetic uncertainty and the initial value variations of kappa and omega on our positive selection results into accounts. For our focal taxa, the phylogenetic uncertainty is mainly related to the phylogenetic position of Scandentia and we respectively used two different phylogenetic relationships for our analyses, ((Scandentia, Primatomorpha), Glires) and ((Scandentia, Glires), Primatomorpha), based on the published studies^63,64^. In addition, to avoid being trapped at a local optimum, we also examined the effects of the initial value variations of kappa and omega on our positive selection results. For this, two different initial values of kappa (kappa = 0.5, 3.0) and two different initial values of omega (omega = 0.5, 2.0) were respectively used for our positive selection analyses, and a total of four independent runs for each positive selected gene were performed. For BS-REL and BUSTED results, when PSGs were identified, a second run was conducted to confirm their positive selection signals. Additionally, to account for the effect of the phylogenetic uncertainty on our results, we used the two different phylogenetic relationships as mentioned above to test the consistency of our results.

### Selection intensity analyses using RELAX

To examine the possible selection intensity changes of dim-light vision, bright-light vision and the motion detection of ancestral primates, we used RELAX^50^, which is available on the Datamonkey webserver (http://test.datamonkey.org/relax), to evaluate the values of the selection intensity parameter (*k*) and its statistical significance of all 33 phototransduction genes that are respectively involved in dim-light vision, bright-light vision and photoresponse recovery. For our analyses, the ancestral primate branch was used as the test branch and all other non-primate branches within Euarchontoglires were used as the reference branches. Given *a priori* partitioned branch sets (e.g., test branches and reference branches) in a codon-based phylogenetic framework, RELAX is capable of determining the selective strength changes, e.g., intensified (*k* > 1) and relaxed (*k* < 1), in the test branches relative to the reference (background) branches using a likelihood ratio test (LRT). Intensified selection is expected to have ω categories away from neutrality (ω = 1), whereas relaxed selection is expected to have ω categories converging to neutrality (ω = 1). LRT is conducted by comparing a null model to an alternative model. In the null model, *k* is constrained to 1 and the same ω distribution on test and background branches is assumed, while in the alternative model, *k* is a free parameter and different ω distributions on test and background branches are assumed. The statistical significance is determined using the standard χ^2^ asymptotic distribution with 1 degree of freedom.

### Ancestral sequence reconstruction and spectral tuning analyses

We reconstructed ancestral amino acid sequences of *LWS* and *SWS1* along branches of interests to examine if there are amino acid replacements that are known to change the spectral tunings of the two opsins. For this, the amino acid-based marginal reconstruction that is implemented in the empirical Bayes approach in PAML 4.8a^69^ was used. For the marginal reconstruction, the character was assigned to one single interior node and the character with the highest posterior probability is considered as the best reconstruction. To test robustness, we used two different amino acid substitution models, JTT and Poisson, for our analyses. The two models have different assumptions about the amino acid substitution rates, with the model JTT assuming different substitution rates of different amino acids while the Poisson model assuming the same substitution rate of all amino acids. As ancestral sequences were reconstructed for internal nodes, the critical amino acid replacements associated with the spectral tunings along branches would be identified. The effects of the amino acid replacements on the spectral tunings of *LWS* and *SWS1* were examined based on previous studies^30,70^ (Supplementary Table 5). Upon analyses, we aligned our sequences against the bovine rhodopsin sequence (NP_001014890) to determine the numbering of amino acids and to identify critical amino acid replacements, and in this study we examined the effects of 10 and 13 critical amino acid replacements of *LWS* and *SWS1*, respectively, on their wavelength shift of maximal absorption (Δλ).

### Protein 3D structure reconstruction

We reconstructed 3D structures of ancestral proteins of two positively selected genes, *ARR3* and *SWS1*, that were identified along ancestral branch of Euarchontoglires. For the structures reconstruction, we firstly reconstructed ancestral sequences of the two genes of the most recent common ancestor of Euarchontoglires using the same approach mentioned above. With the reconstructed ancestral sequences, SWISS-MODEL (https://swissmodel.expasy.org/) was employed to reconstruct the 3D structures. SWISS-MODEL is an automated web-based system that uses homology modeling techniques for modeling the 3D structure of a protein based on its amino acid sequence, and typically using the amino acid sequence of the target protein to search for the most suitable template, which is the experimentally determined 3D structure, and then a homology model is built accordingly with the quality estimation (GMQE value)^71^. For our analyses, the most suitable templates for our focal proteins of *ARR3* and *SWS1* were determined to be 1suj.1.A with a GMQE value of 0.82 and 5dys.1.A with a GMQE value of 0.74, respectively. Upon the templates selected, the corresponding homology 3D structures were subsequently built and visualized and modified using PyMOL (The PyMOL Molecular Graphics System, Version 1.3, Schrödinger LLC, https://www.pymol.org/).

## Electronic supplementary material

Supplementary Files

Supplementary Table 1
