# Supplementary material for: Rethinking the Origin of Primates by Reconstructing Their Diel Activity Patterns Using Genetics and Morphology
Source: Sci Rep. 2017 Sep 19;7:11837. doi: 10.1038/s41598-017-12090-3 (PMC5605515; doi:10.1038/s41598-017-12090-3)
Supplement: Supplementary file 1 — Supplementary Files [file 41598_2017_12090_MOESM1_ESM.doc]

**Rethinking the Origin of Primates by Reconstructing Their Diel Activity Patterns Using Genetics and Morphology**

### Yonghua Wu, Haifeng Wang, Haitao Wang, [Elizabeth A. Hadly](http://web.stanford.edu/group/hadlylab/people/hadly.html)

| **Supplementary Files**  **Supplementary Figure 1.** Genes involved in cones/rods phototransduction pathway.  **Supplementary Figure 2.** *ARR3* amino acids alignment.  **Supplementary Figure 3.** *SWS1* amino acids alignment.  **Supplementary Figure 4.** Positively selected amino acid sites of *SWS1* mapping on the secondary structure of bovine rhodopsin.  **Supplementary Figure 5.** Comparisons of the reconstructed diel activity patterns in primates.  **Supplementary Figure 6.** The critical amino acid replacements associated with *LWS* spectral tuning.  **Supplementary Figure 7.** *LWS* amino acid alignment.  **Supplementary Table 2.** Positively selected sites identified based on the branch-site model.  **Supplementary Table 3.** Positively selected genes identified by BUSTED.  **Supplementary Table 4.** Positively selected genes identified by BS-REL.  **Supplementary Table 5.** Amino acid replacements of visual opsins (*LWS* and *SWS1*) and their effects on the wavelength shift of maximal absorption (Δλ). |
| --- |
|  |
| 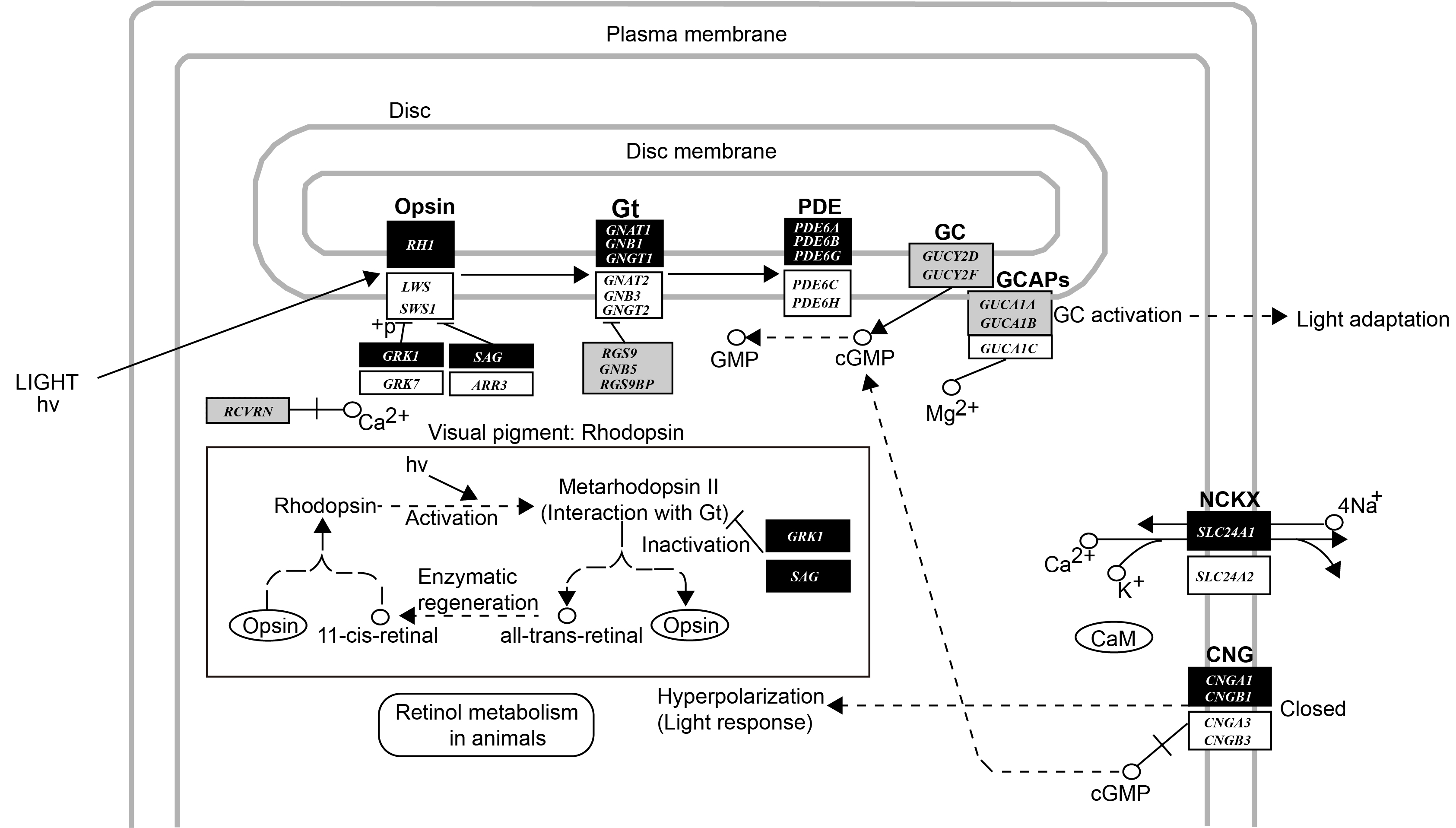 |

**Supplementary Figure 1** **Phototransduction pathway in rods (according to KEGG pathway: map04744) and the phototransduction genes used in this study (italicized).** For convenience, the genes involved in the phototransduction pathway in cones are also shown. Dark rectangles, white rectangles and grey rectangles show genes that are involved in the phototransduction pathway of rods, cones and both, respectively . The phototransduction signalling begins with the activation of opsins by light, and the activated opsins subsequently activate their downstream protein, G-protein (Gt). Gt, in turn, stimulates the cGMP phosphodiesterase (PDE6), which then hydrolyzes cGMP to GMP, resulting in closure of cyclic nucleotide-gated (CNG) channels and hyperpolarizing the cell. The closure of CNG channels reduces cytoplasmic Ca^2+^ concentration by a Na^+^/Ca^2+^-K^+^ exchanger (NCKX), and helps to restores the cGMP concentration through activating guanylyl cyclase activating proteins (GCAPs) and hence guanylyl cyclase (GC), leading to the re-opening of CNG channels. The re-opening of CNG channels is important for photoresponse recovery. For photoresponse recovery, other molecular components are also involved. For instance, *GRK1* and *SAG* are known to inactivate the activated rhodopsin (*RH1*), and *GRK7* and *ARR3* are known to inhibit cone opsins (e.g., *LWS* and *SWS1*). In addition, *RGS9*, *GNB5* and *RGS9BP* are known to work as a complex to inhibit activated Gt.


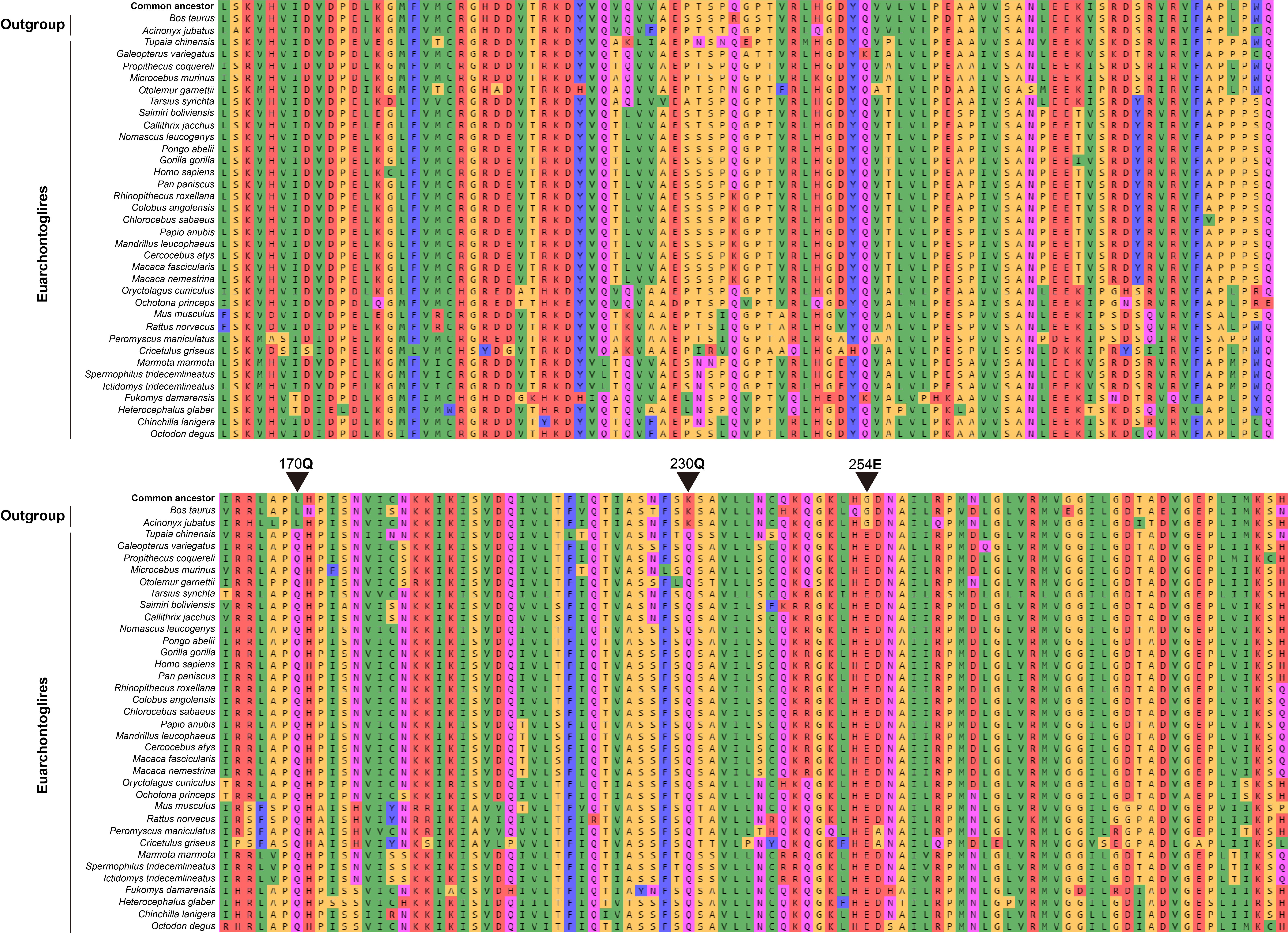


**Supplementary Figure 2** ***ARR3* amino acids alignment and positively selected sites.** For the alignment, only variable sites are shown here. Three positively selected sites are shown in arrows. The three positively selected sites show fixed amino acid differences between Euarchontoglires and outgroup.


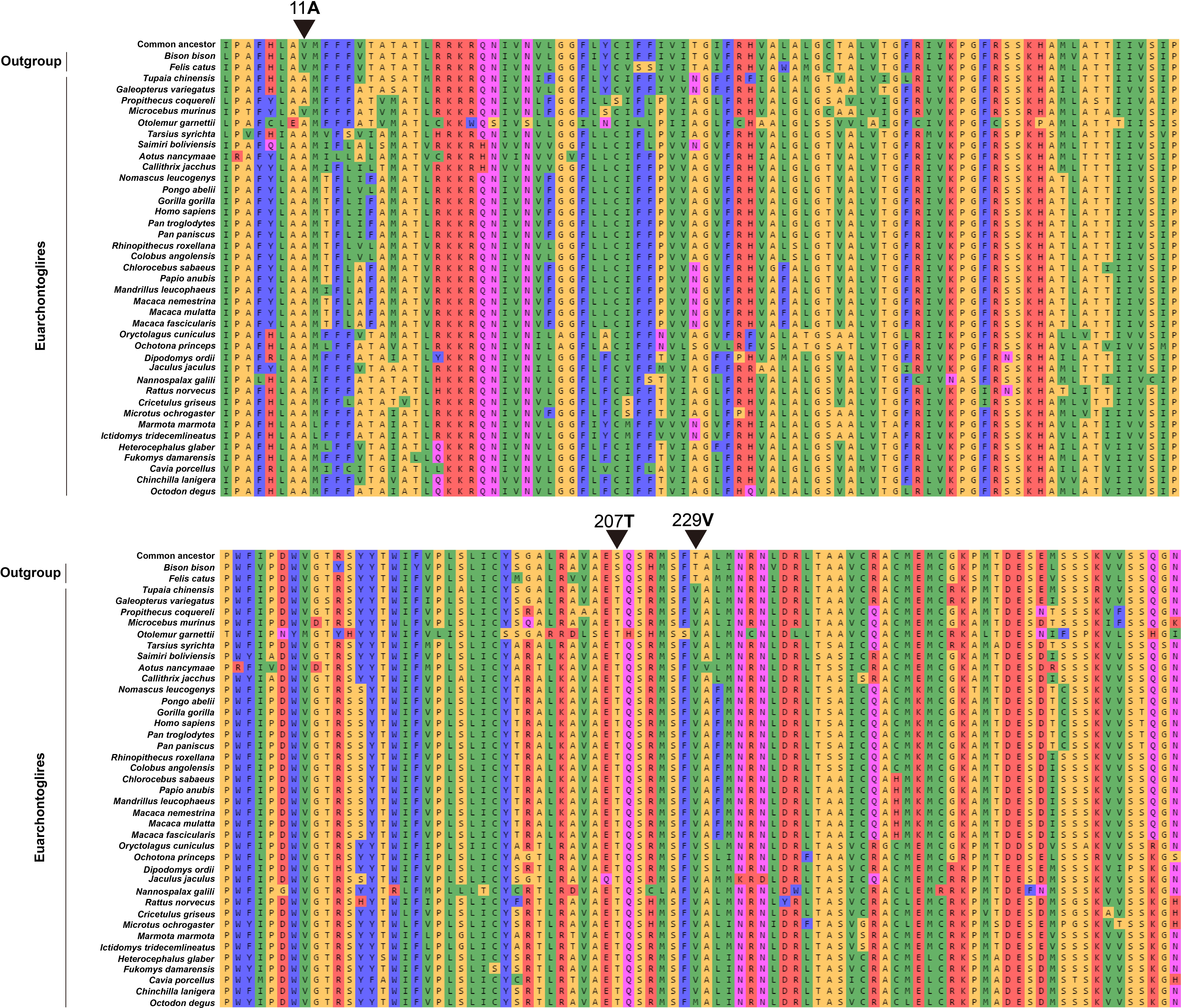


**Supplementary Figure 3** ***SWS1* amino acids alignment and positively selected sites.** For the alignment, only variable sites are shown here. Three positively selected sites are shown in arrows. The three positively selected sites show fixed (207T) or nearly fixed (11A and 229V) amino acid differences between Euarchontoglires and outgroup.


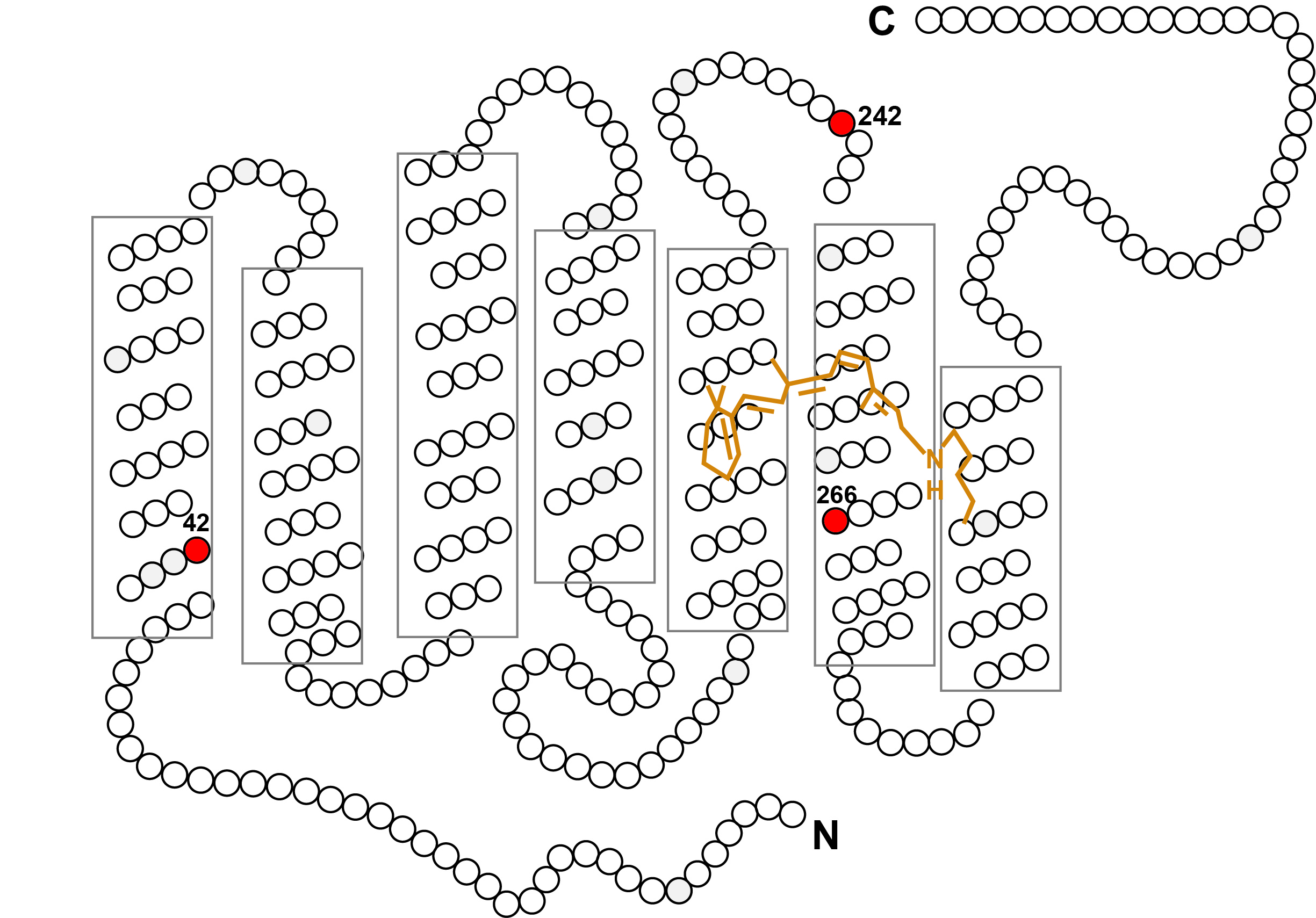


**Supplementary Figure 4** **Positively selected amino acid sites of *SWS1* mapping on the secondary structure of bovine rhodopsin.** Three positively selected sites of *SWS1*, 11A, 207T and 229V, that correspond to the three sites (42, 242 and 266) of the bovine rhodopsin are shown in red. Seven transmembrane domains of rhodopsin are shown in grey rectangles, and the 11-cis-retinal is shown in orange. The secondary structure of the bovine rhodopsin is based on previous studies .


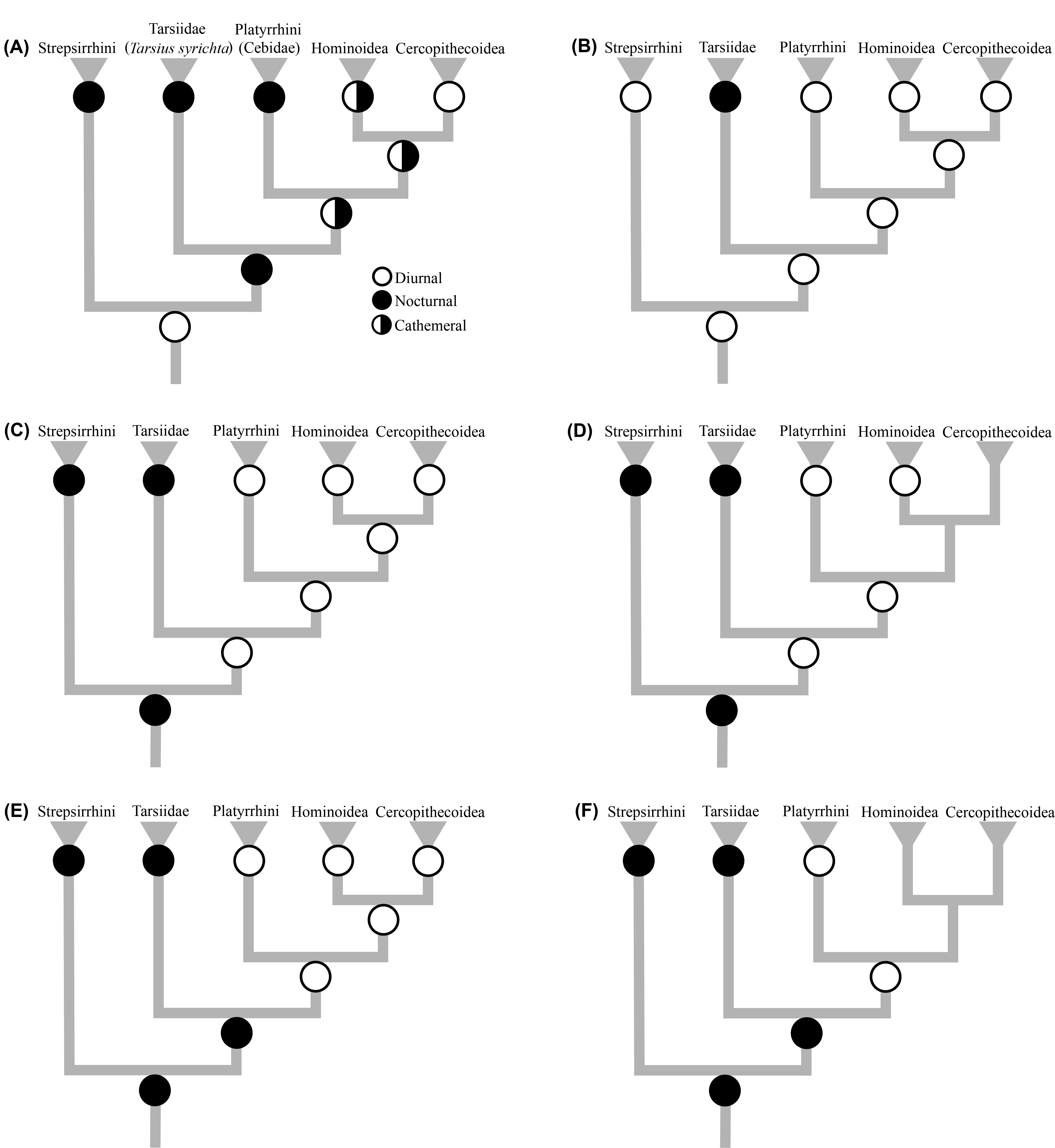


**Supplementary Figure 5** **Comparisons of the reconstructed diel activity patterns in primates**. The reconstructed results of this study are shown in (**A**), and the results of other previous studies are shown in (**B**) ^16^, (**C**) ^10^, (**D**) ^11^, (**E**) ^8^ and (**F**) ^9^. Noting that, for our results (**A**), only one species (*Tarsius syrichta*) from Tarsiidae was involved, and only three species of Cebidae (*Callithrix jacchus*, *Aotus nancymaae* and *Saimiri boliviensis* ) from Platyrrhini were involved, and hence their corresponding diel activity patterns inferred here would not represent that of ancestral Tarsiidae and ancestral Platyrrhini, respectively.


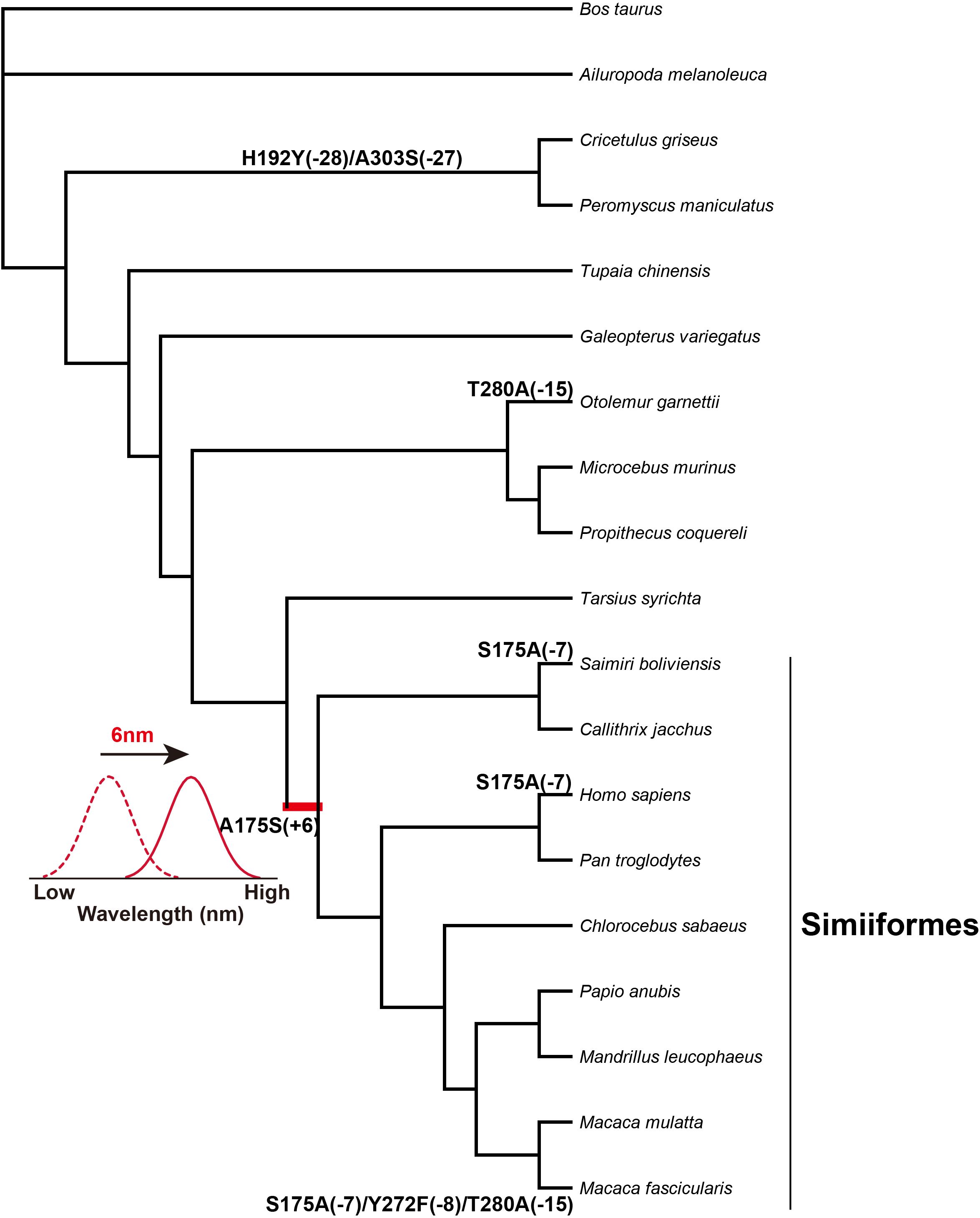


**Supplementary Figure 6 The critical amino acid replacements associated with *LWS* spectral tuning along different branches.** The effects of the critical amino acid replacements on *LWS* spectral tuning follow previous studies (Table S5), and here are shown in parenthesis. For ancestral simian primate branch, the amino acid replacement, A175S, is known to increase λmax of *LWS* by 6nm ^30^. The phylogenetic relationships among species follow previous studies . Ancestral amino acid sequences of the internal nodes were reconstructed based on the empirical Bayes approach using the JTT model of the amino acid substitution.


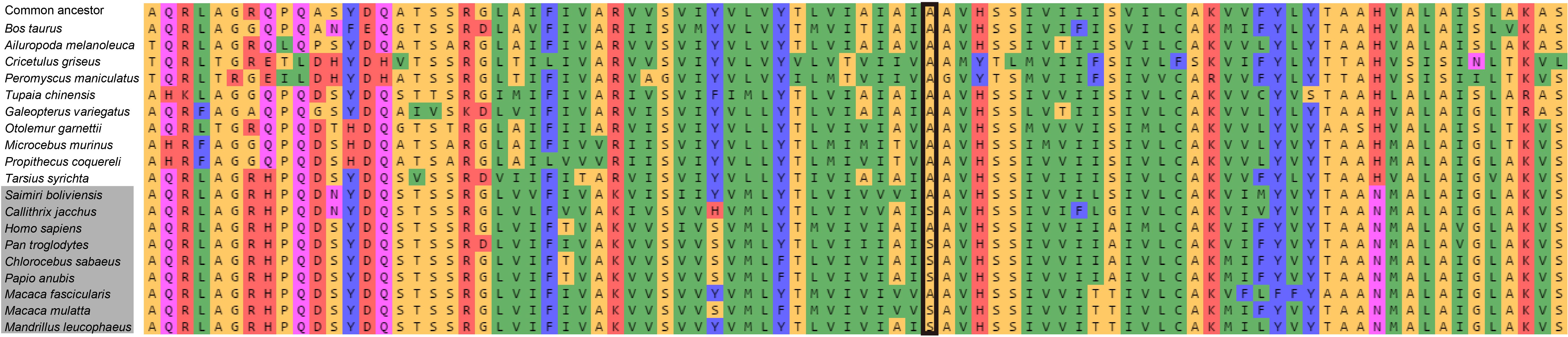


**Supplementary Figure 7 *LWS* amino acid alignment.** For alignment, only variable sites are shown. The critical amino acid replacement (A175S) associated with *LWS* spectral tuning along ancestral branch of Simiiformes (grey area) is highlighted in black.

**Supplementary Table 2 Positively selected sites identified based on the branch-site model of PAML.** The sites with >95% posterior probabilities support are shown in bold.

| **Taxa /Genes** | **Positively selected sites** |
| --- | --- |
| **Euarchontoglires** |  |
| *ARR3* | 170Q, 230Q, 254E  230 Q 0.601  254 E 0.912 |
|  |  |
| *SWS1* | 11A, 207T, **229V** |
|  |  |
| **Dermoptera** |  |
| *CNGA1* | 79S, 91T, 92L, **150E**, **204Q**, 215V, 293Y, **525G**, 547R, 550G, 595E |
|  |  |
| *PDE6H* | 6L |
|  |  |
| **Strepsirrhini** |  |
| *GNGT1* | **35C** |
|  |  |
| **Haplorrhini** |  |
| *CNGB1* | 88Q, **102Q**, 157L, 223C, 234L, 297W |
|  |  |
| **Tarsiidae** |  |
| *PDE6B* | **158P**, 627A |
|  |  |
| **Simiiformes** |  |
| *GRK1* | 70R, **238A** |
|  |  |
| *SWS1* | 196R, 296S |
|  |  |
| **Platyrrhini** |  |
| *PDE6B* | 32V, 43D, 51C, **85T**, 636M |
|  |  |
| **Cercopithecidae** |  |
| *CNGB3* | 47L, 116H, **236S** |
|  |  |
| **Aotus** |  |
| *RCVRN* | 113G |
|  |  |
| **Glires** |  |
| *GNGT2* | 9E, 25P, 65G |
|  |  |

| **Supplementary Table 3 Positively selected genes identified by BUSTED.** For analyses, each taxon was respectively treated as foreground branch (FG) and others were used as background branches (BG). Only branches of interest with positively selected genes found are shown. | | | | | | | | | | | |
| --- | --- | --- | --- | --- | --- | --- | --- | --- | --- | --- | --- |
| **Taxa/Gene** | **Model** | ***log* L** | **#par.** | **Branch**  **set** | **ω1** |  | **ω2** |  | **ω3** | ***P*-value** | **Corrected**  ***P*-value** |
|  |  |  |  |  |  | |  | |  |  |  |
| **Primates** |  |  |  |  |  | |  | |  |  |  |
| *PDE6C* | Unconstrained | -20456.04 | 99 | BG | 0.114 (93%) | | 0.117 (5.6%) | | 4.66 (1.5%) |  |  |
|  |  |  |  | FG | 0.0664 (95%) | | 0.468 (5.2%) | | **10000 (0.30%)** | 0.0102 | 0.1734 |
|  | Constrained | -20460.62 | 98 | BG | 0.115 (93%) | | 0.118 (5.4%) | | 4.78 (1.4%) |  |  |
|  |  |  |  | FG | 0.00 (54%) | | 0.00 (21%) | | 1.00 (25%) |  |  |
| *SWS1* | Unconstrained | -8550.16 | 101 | BG | 0.145(95%) | | 0.151(3.4%) | | 10.7(1.4%) |  |  |
|  |  |  |  | FG | 0.00 (87%) | | 0.00 (7.5%) | | **30.9 (5.3%)** | 0.0113 | 0.1921 |
|  | Constrained | -8554.65 | 100 | BG | 0.144 (95%) | | 0.170 (3.6%) | | 10.7 (1.4%) |  |  |
|  |  |  |  | FG | 0.00 (23%) | | 0.00 (19%) | | 1.00 (58%) |  |  |
| *PDE6A* | Unconstrained | -18940.73 | 105 | BG | 0.0459 (96%) | | 1.00 (4.2%) | | 40.4 (0.045%) |  |  |
|  |  |  |  | FG | 0.0913 (96%) | | 0.168 (4.2%) | | **1680 (0.17%)** | 0.031 | 0.527 |
|  | Constrained | -18944.21 | 104 | BG | 0.0447 (96%) | | 1.00 (4.3%) | | 38.4 (0.043%) |  |  |
|  |  |  |  | FG | 0.0130 (0.0%) | | 0.00 (87%) | | 1.00 (13%) |  |  |
| **Euarchontoglires** |  |  |  |  |  |  |  |  |  |  |  |
| *CNGB3* | Unconstrained | -15570.26 | 99 | BG | 0.0315 (70%) | | 0.696 (21%) | | 2.35 (9.7%) |  |  |
|  |  |  |  | FG | 0.00 (74%) | | 1.00 (25%) | | **136 (0.51%)** | 0.0438 | 0.657 |
|  | Constrained | -15573.39 | 98 | BG | 0.0306 (70%) | | 0.707 (21%) | | 2.35 (9.6%) |  |  |
|  |  |  |  | FG | 0.00 (73%) | | 0.925 (0.0%) | | 1.00 (27%) |  |  |
| *SWS1* | Unconstrained | -8552.04 | 101 | BG | 0.144 (95%) | | 0.141 (3.0%) | | 10.8 (1.5%) |  |  |
|  |  |  |  | FG | 0.127 (87%) | | 0.576 (9.8%) | | **27.6 (3.1%)** | 0.0505 | 0.8585 |
|  | Constrained | -8555.03 | 100 | BG | 0.145 (95%) | | 0.134 (3.1%) | | 11.0 (1.5%) |  |  |
|  |  |  |  | FG | 0.00 (58%) | | 0.923 (0.0%) | | 1.00 (42%) |  |  |
| **Haplorrhini** |  |  |  |  |  | |  | |  |  |  |
| *CNGB1* | Unconstrained | -14896.42 | 95 | BG | 0.0346 (94%) | | 1.00 (5.9%) | | 23.8 (0.080%) |  |  |
|  |  |  |  | FG | 0.409 (87%) | | 1.00 (12%) | | **534 (0.62%)** | 0.0042 | 0.0672 |
|  | Constrained | -14901.90 | 94 | BG | 0.0329 (94%) | | 0.993 (6.1%) | | 24.4 (0.079%) |  |  |
|  |  |  |  | FG | 0.00 (74%) | | 0.928 (0.0%) | | 1.00 (26%) |  |  |
| *CNGB3* | Unconstrained | -15568.12 | 99 | BG | 0.0351 (70%) | | 0.712 (20%) | | 2.34 (9.4%) |  |  |
|  |  |  |  | FG | 0.00 (84%) | | 0.00 (12%) | | **59.7 (4.4%)** | 0.0107 | 0.1605 |
|  | Constrained | -15572.65 | 98 | BG | 0.0333 (70%) | | 0.719 (20%) | | 2.36 (9.3%) |  |  |
|  |  |  |  | FG | 1.00 (3.4%) | | 0.00 (12%) | | 1.00 (84%) |  |  |
| *SLC24A2* | Unconstrained | -14332.96 | 95 | BG | 0.104 (96%) | | 0.0985 (2.6%) | | 3.86 (0.99%) |  |  |
|  |  |  |  | FG | 0.481 (93%) | | 1.00 (6.4%) | | **1470 (0.29%)** | 0.0239 | 0.3585 |
|  | Constrained | -14336.70 | 94 | BG | 0.103 (96%) | | 0.124 (2.6%) | | 3.96 (0.96%) |  |  |
|  |  |  |  | FG | 1.00 (25%) | | 0.911 (0.0%) | | 1.00 (75%) |  |  |
| **Simiiformes** |  |  |  |  |  | |  | |  |  |  |
| *CNGA1* | Unconstrained | -16354.60 | 103 | BG | 0.00210 (76%) | | 0.485 (24%) | | 16.5 (0.26%) |  |  |
|  |  |  |  | FG | 0.00 (87%) | | 0.00 (8.6%) | | **11.8 (4.3%)** | 0.0084 | 0.1428 |
|  | Constrained | -16359.39 | 102 | BG | 0.0200 (82%) | | 0.575 (18%) | | 15.6 (0.27%) |  |  |
|  |  |  |  | FG | 0.00 (76%) | | 0.00 (1.5%) | | 1.00 (23%) |  |  |
| *GNB3* | Unconstrained | -6133.60 | 103 | BG | 0.0163 (96%) | | 0.0451 (3.6%) | | 5.81 (0.25%) |  |  |
|  |  |  |  | FG | 0.0770 (91%) | | 0.103 (8.6%) | | **80.2 (0.89%)** | 0.0253 | 0.4301 |
|  | Constrained | -6137.27 | 102 | BG | 0.0164 (96%) | | 0.0438 (3.7%) | | 5.77 (0.25%) |  |  |
|  |  |  |  | FG | 0.0315 (0.0%) | | 0.00 (86%) | | 1.00 (14%) |  |  |
| *RGS9BP* | Unconstrained | -6601.80 | 91 | BG | 0.0586 (90%) | | 0.154 (8.6%) | | 2.88 (1.4%) |  |  |
|  |  |  |  | FG | 0.316 (82%) | | 0.329 (14%) | | **33.2 (3.2%)** | 0.0403 | 0.5642 |
|  | Constrained | -6605.01 | 90 | BG | 0.0616 (90%) | | 0.121 (8.7%) | | 2.84 (1.4%) |  |  |
|  |  |  |  | FG | 1.00 (8.9%) | | 0.00 (62%) | | 1.00 (29%) |  |  |
| **Catarrhini** |  |  |  |  |  | |  | |  |  |  |
| *LWS* | Unconstrained | -5040.24 | 59 | BG | 0.0698 (93%) | | 0.0807 (5.2%) | | 4.41 (2.0%) |  |  |
|  |  |  |  | FG | 0.0563 (93%) | | 0.0661 (6.3%) | | **10000 (0.44%)** | 0.0273 | 0.4095 |
|  | Constrained | -5043.84 | 58 | BG | 0.0695 (93%) | | 0.0953 (5.1%) | | 4.47 (2.0%) |  |  |
|  |  |  |  | FG | 0.0191 (0.0%) | | 0.00 (89%) | | 1.00 (11%) |  |  |
| **Cercopithecidae** |  |  |  |  |  |  |  |  |  |  |  |
| *RH1* | Unconstrained | -8140.16 | 109 | BG | 0.0204 (97%) | | 0.844 (2.8%) | | 263 (0.13%) |  |  |
|  |  |  |  | FG | 0.00 (96%) | | 0.00 (3.5%) | | **40.4 (0.63%)** | 0.0049 | 0.0833 |
|  | Constrained | -8145.48 | 108 | BG | 0.0197 (97%) | | 0.791 (3.1%) | | 255 (0.13%) |  |  |
|  |  |  |  | FG | 0.00 (96%) | | 0.00 (0.27%) | | 1.00 (3.6%) |  |  |
| **Scandentia** |  |  |  |  |  |  |  |  |  |  |  |
| *PDE6A* | Unconstrained | -18932.34 | 105 | BG | 0.0404 (95%) | | 1.00 (4.6%) | | 267 (0.017%) |  |  |
|  |  |  |  | FG | 0.0822 (87%) | | 0.799 (12%) | | **49.3 (0.53%)** | 0.0424 | 0.7208 |
|  | Constrained | -18935.50 | 104 | BG | 0.0406 (95%) | | 0.973 (4.6%) | | 76.2 (0.028%) |  |  |
|  |  |  |  | FG | 0.00 (80%) | | 0.928 (0.0%) | | 1.00 (20%) |  |  |
| **Glires** |  |  |  |  |  | |  | |  |  |  |
| *GNGT2* | Unconstrained | -1579.01 | 73 | BG | 0.0698 (82%) | | 0.104 (11%) | | 1.84 (7.3%) |  |  |
|  |  |  |  | FG | 0.885 (88%) | | 1.00 (9.0%) | | **348 (3.1%)** | 0.0461 | 0.461 |
|  | Constrained | -1582.09 | 72 | BG | 0.0720 (82%) | | 0.0875 (11%) | | 1.82 (7.4%) |  |  |
|  |  |  |  | FG | 0.00 (61%) | | 0.924 (0.0%) | | 1.00 (39%) |  |  |
| *GRK7* | Unconstrained | -12727.12 | 87 | BG | 0.0257 (79%) | | 0.861 (8.5%) | | 1.03 (13%) |  |  |
|  |  |  |  | FG | 0.522 (87%) | | 0.979 (12%) | | **278 (0.58%)** | 0.0382 | 0.6494 |
|  | Constrained | -12730.38 | 86 | BG | 0.00 (74%) | | 0.863 (26%) | | 0.871 (0.0%) |  |  |
|  |  |  |  | FG | 0.00 (51%) | | 0.845 (0.0%) | | 1.00 (49%) |  |  |
| *SLC24A1* | Unconstrained | -29514.13 | 105 | BG | 0.0319 (60%) | | 0.946 (29%) | | 1.18 (11%) |  |  |
|  |  |  |  | FG | 0.394 (91%) | | 0.984 (8.3%) | | **1780 (0.31%)** | 0.0113 | 0.1921 |
|  | Constrained | -29518.61 | 104 | BG | 0.0179 (58%) | | 0.984 (37%) | | 1.07 (4.8%) |  |  |
|  |  |  |  | FG | 0.00 (52%) | | 0.884 (0.0%) | | 1.00 (48%) |  |  |
| **Rodents** |  |  |  |  |  | |  | |  |  |  |
| *CNGA3* | Unconstrained | -12302.10 | 109 | BG | 0.0149 (88%) | | 0.444 (8.4%) | | 1.09 (3.3%) |  |  |
|  |  |  |  | FG | 0.00 (95%) | | 0.00 (4.4%) | | **10000 (0.41%)** | 0.0074 | 0.1258 |
|  | Constrained | -12307.00 | 108 | BG | 0.0226 (90%) | | 0.406 (6.9%) | | 1.22 (3.2%) |  |  |
|  |  |  |  | FG | 0.00 (24%) |  | 0.00(70%) |  | 1.00(6.2%) |  |  |
| *RCVRN* | Unconstrained | -4837.35 | 107 | BG | 0.0903 (95%) | | 0.0955 (4.3%) | | 3.97 (0.59%) |  |  |
|  |  |  |  | FG | 0.00 (93%) | | 0.00 (5.5%) | | **10000 (1.0%)** | 0.0014 | 0.0238 |
|  | Constrained | -4843.95 | 106 | BG | 0.0873 (94%) | | 0.0932 (5.4%) | | 3.84 (0.75%) |  |  |
|  |  |  |  | FG | 1.00 (10%) | | 0.00518 (0.0%) | | 1.00 (90%) |  |  |
| *PDE6B* | Unconstrained | -18164.39 | 101 | BG | 0.0444 (96%) | | 0.0887 (2.8%) | | 3.69 (1.1%) |  |  |
|  |  |  |  | FG | 0.0111 (95%) | | 0.00 (4.7%) | | **63.6 (0.72%)** | 0.0493 | 0.7395 |
|  | Constrained | -18167.40 | 100 | BG | 0.0246 (95%) | | 1.00 (4.8%) | | 44.8 (0.068%) |  |  |
|  |  |  |  | FG | 0.00 (94%) | | 0.00 (0.38%) | | 1.00 (5.8%) |  |  |

log L,  log-likelihood values, # par., the number of parameters, Corrected *P*-value, Bonferroni multiple testing correction, P values are corrected by multiply them by the number of branches tested of each gene. Significance level is *P*<0.05.

**Supplementary Table 4 Positively selected genes identified by BS-REL.** Only branches of interest with positively selected genes found (corrected *P* value <0.05) are shown.

| **Taxa/gene** | **Mean ω** | **ω1** | **p1** | **ω2** | **p2** | **ω3** | **p3** | **LRT** | ***P*-value** | **Corrected** |
| --- | --- | --- | --- | --- | --- | --- | --- | --- | --- | --- |
|  |  |  |  |  |  |  |  |  |  | ***P*-value** |
|  |  |  |  |  |  |  |  |  |  |  |
| **Haplorrhini** |  |  |  |  |  |  |  |  |  |  |
| *CNGB1* | 0.946154 | 0.33 | 0.983705 | 0.397004 | 0.009403 | 370.279 | 0.006892 | 11.0829 | 0.000436 | 0.030931 |
| **Cercopithecidae** |  |  |  |  |  |  |  |  |  |  |
| *RH1* | 0.085559 | 0 | 0.988445 | 0 | 0.00526 | 42.2093 | 0.006295 | 10.9692 | 0.000463 | 0.03752 |
| **Rodents** |  |  |  |  |  |  |  |  |  |  |
| *CNGA3* | 0.040351 | 0 | 0.994467 | 0 | 0.001385 | 9611.73 | 0.004148 | 11.4337 | 0.000361 | 0.030652 |
| *PDE6B* | 0.070127 | 0 | 0.989522 | 0 | 0.001985 | 61.9185 | 0.008493 | 14.2521 | 7.99E-05 | 0.006075 |
|  |  |  |  |  |  |  |  |  |  |  |

p1, p2 and p3 are proportion of sites classified to ω1, ω2 and ω3, LRT: likelihood ratio test statistic, corrected *P* value: after an application of Holm’s multiple testing correction.

**Supplementary Table 5 Amino acid replacements of visual opsins (*LWS* and *SWS1*) and their effects on the wavelength shift of maximal absorption (Δλ)** . Amino acid site numbers are based on the bovine rhodopsin.

| **Opsin** | **Amino acid replacement** | **∆λ (nm)** |
| --- | --- | --- |
|  |  |  |
| ***LWS*** |  |  |
|  | S164A | -7 |
|  | A164S | +6 |
|  | H181Y | -28 |
|  | Y261F | -8 |
|  | F261Y | +6 |
|  | T269A | -15 |
|  | A269T | +10 |
|  | A292S | -27 |
|  | S292A | +28 |
|  | S164A & H181Y | +11 |
| ***SWS1*** |  |  |
|  | F86Y | +66 |
|  | Y86F | -75 |
|  | F86S | +17 |
|  | S86F | -52 |
|  | S90G | -7 |
|  | S90C | -7 |
|  | C90S | +38 |
|  | I93T | -6 |
|  | E113D | -4 |
|  | D113E | -12 |
|  | V116L | -3 |
|  | A118T | +3 |
|  | Y265W | +10 |
